# Early transmission of sensitive strain slows down emergence of drug resistance in *Plasmodium vivax*

**DOI:** 10.1371/journal.pcbi.1007945

**Published:** 2020-06-17

**Authors:** Mario J. C. Ayala, Daniel A. M. Villela

**Affiliations:** Programa de Computação Científica, Fundação Oswaldo Cruz (Fiocruz), Rio de Janeiro, Brazil; The University of Melbourne Melbourne School of Psychological Sciences, AUSTRALIA

## Abstract

The spread of drug resistance of *Plasmodium falciparum* and *Plasmodium vivax* parasites is a challenge towards malaria elimination. *P. falciparum* has shown an early and severe drug resistance in comparison to *P. vivax* in various countries. In fact, *P. vivax* differs in its life cycle and treatment in various factors: development and duration of sexual parasite forms differ, symptoms severity are unequal, relapses present only in *P. vivax* cases and the Artemisinin-based combination therapy (ACT) is only mandatory in *P. falciparum* cases. We compared the spread of drug resistance for both species through two compartmental models using ordinary differential equations. The model structure describes how sensitive and resistant parasite strains infect a human population treated with antimalarials. We found that an early transmission,i.e., before treatment and low effectiveness of drug coverage, supports the prevalence of sensitive parasites delaying the emergence of resistant *P. vivax*. These results imply that earlier attention of both symptomatic cases and reservoirs of *P. vivax* are essential in controlling transmission but also accelerate the spread of drug resistance.

## Author summary

The main strategy to treat and prevent malaria still relies on the use of drugs targeting *Plasmodium falciparum* and *Plasmodium vivax* parasites. Since the worldwide number of cases is still large, there is a risk of emergence of drug resistance, which is precisely the case of resistance of chloroquine for *P. falciparum* and *P. vivax* in some countries. The spread of drug resistance challenges malaria control programs due to treatment failures and consequent lower drug efficacy. This phenomenon is more frequent, accelerated, and studied in *P. falciparum* cases but *P. vivax* also presents drug resistance in some areas and the particular life cycle of *P. vivax* with relapses due to its latent form, an earlier transmission and more common low parasitemia densities that imposes detection difficulties. We developed a mathematical model to study drug-resistance emergence in *P. vivax* finding that early transmission of drug-sensitive parasites as well as infected humans without symptoms delays drug resistance. As a consequence, however, the number of *P. vivax* cases decreases less than the number of *P. falciparum* cases. These results suggest that earlier attention of all infected humans, including individuals without symptoms, might help to decrease *P. vivax* cases but also accelerate the spread of drug resistance.

## Introduction

The World Health Organization (WHO) estimated 219 million cases of malaria in 2017, most of them caused by *Plasmodium falciparum* due to high presence in Africa, with 96.6% of total numbers [1]. However, *P. falciparum* does not maintain such high dominance in other continents, since *Plasmodium vivax* is diagnosed in 74.1% and 37.2% of cases in the Americas and Southeast Asia, respectively [1]. Understanding conditions that drive the emergence of drug resistance for these species is vital in the goal of ending epidemics of malaria by 2030 in the Sustainable Development Goals (SDG 3.3) in United Nations [2].

Antimalarials have been the main strategy for controlling transmission, but drug resistance has emerged to drugs for *P. falciparum* infection implying slower clearance rates and treatment failures [3–5]. Currently, Artemisinin-based combination therapy (ACT) has constituted first-line treatment for *P. falciparum* as a fast-acting artemisinin derivative plus a longer-acting partner drug [6]. However, previous studies found resistance levels and slow clearance rates using ACTs such as dihydroartemisinin-piperaquine and artesunate-mefloquine in the Greater Mekong Subregion, bringing a need for developing new treatments [7–10].

*P. vivax* and *P. falciparum* differ in their life cycles [11–13] because *P. vivax* has a set of particularities that challenge malaria control: development in temperate climates, production of dormant-stage parasite forms (hypnozoites), development of low parasitemia densities and earlier transmission of sexual-parasite forms [14]. By contrast, although drug resistance in *P. vivax* also challenges malaria control programs, chloroquine (CQ) remains as first-line treatment for *P. vivax* while the extended CQ use for *P. falciparum* spread CQ-resistance for this parasite worldwide [6, 15, 16]. Nevertheless, CQ-resistance in *P. vivax* already affects some regions inducing the adoption of ACTs. Also, low parasitemia densities impose detection difficulties, which contribute to underestimating the real impact of drug resistance in *P. vivax* [11, 17]. Previous works have compared *P. vivax* with *P. falciparum* resistance through in vivo, in vitro, and molecular assays to test resistance, drug susceptibility, and characterization of gene changes [18]. However, such studies for *P. vivax* present difficulties in replicating the life cycle, producing a knowledge gap in understanding drug resistance in this species [19, 20].

Mathematical models provide an understanding of drug resistance to guide the development of malaria programs [21], through both deterministic and stochastic models simulating the implementation of monotherapies and combination therapies [22]. Models also showed the contribution on the emergence of resistant strains due to factors: fitness cost, selection of resistant parasites after treatment, changes in transmission settings, efficiency in drug dose and the role of asymptomatics [21–35]. These previous works inferred that sub-optimal doses, high treatment coverage, and lower levels of immunity have a direct relation to drug resistance. However, they based their findings in *P. falciparum* life cycle avoiding the particular features of *P. vivax*, producing an inaccurate extension of model results in the case of *P. vivax* control programs.

Previous *P. vivax* models focused on exploring the effect of relapses on malaria prevalence, and Schneider and Scalante placed a feature in their model to consider the evolution of drug resistance [36] by assessing parasite selection on a genetic model. Previous works estimated that relapses cause around 80% of *P. vivax* cases in children in Papua New Guinea suggesting a high epidemiological effect of hypnozoites [37, 38]. This finding agrees with previous models that predict a greater *P. vivax* prevalence than *P. falciparum* in the same transmission settings due to relapses [39] and a direct relation between *P. vivax* prevalence and relapse frequency [40]. Elimination programs must consider a relapse treatment with primaquine (PQ). Also, previous works have inferred that a potent treatment with ACTs plus primaquine (PQ) would eliminate *P. vivax* in low transmission settings by implementing programs of mass drug administration [41–43]. Nevertheless, *P. vivax* demonstrated an unstable elimination environment, when compared to *P. falciparum* [41], since PQ triggers an adverse effect to individuals affected by glucose-6-phosphate-dehydrogenase deficiency (G6PD) questioning mass programs [40, 44]. Previous works also indicated that the early development of gametocytes increases *P. vivax* incidence [40, 45].

We aim to study the emergence and spread of *P. vivax* and *P. falciparum* drug resistance taking into account *P. vivax* particularities: relapses, earlier transmission, and detection difficulties due to asymptomatic cases. We developed compartmental models for both *P. vivax* and *P. falciparum* illustrating the emergence and transmission of one resistant strain on a wild-strain population under the pressure of treatments with CQ and ACTs plus addition or no PQ. We implemented equivalent epidemiological settings for human and mosquito populations to make comparable drug-resistance evolution between *Plasmodium* species. Our approach reveals the impact of *P. vivax* particularities in drug resistance filling in the gap of knowledge about *P. vivax* resistance.

## Materials and methods

We developed mathematical models for both *Plasmodium vivax* and *Plasmodium falciparum* using ordinary differential equations (ODE) to represent the transmission of two strains: sensitive and resistant. The fundamentals from these models have origin on the well-known Ross-Macdonald model that separates human and mosquito populations by susceptible and infected individuals [46]. Additionally, we implemented a post-treatment state in humans, and we also distinguished infected states by sensitive and resistant strains.

The model structure considers only a single genotype per infected human, either sensitive or resistant. However, multiple malaria parasites with different genotypes might infect humans. Multiple-genotype infections can happen due to genetically distinct sporozoites, either from a single inoculation (co-inoculation) or multiple inoculations (superinfection). The relative importance of co-inoculation versus superinfection is setting dependent and not yet fully understood. Previous studies found that parasite density in blood-stage can limit subsequent development of new sporozoites supporting the parasite population of first-inoculated genotype [47, 48]. Hence, superinfection may be limited. Another study analyzing multiple-genotype infections found multiple parasite haploids with genetic similarity suggesting infection from a single inoculation rather than several inoculations [49]. The model presented here uses a single genotype per infected human for model simplification.

The next subsections expand model features and differences between *P. vivax* and *P. falciparum* modeling.

### *P. falciparum* model

**Fig 1:**
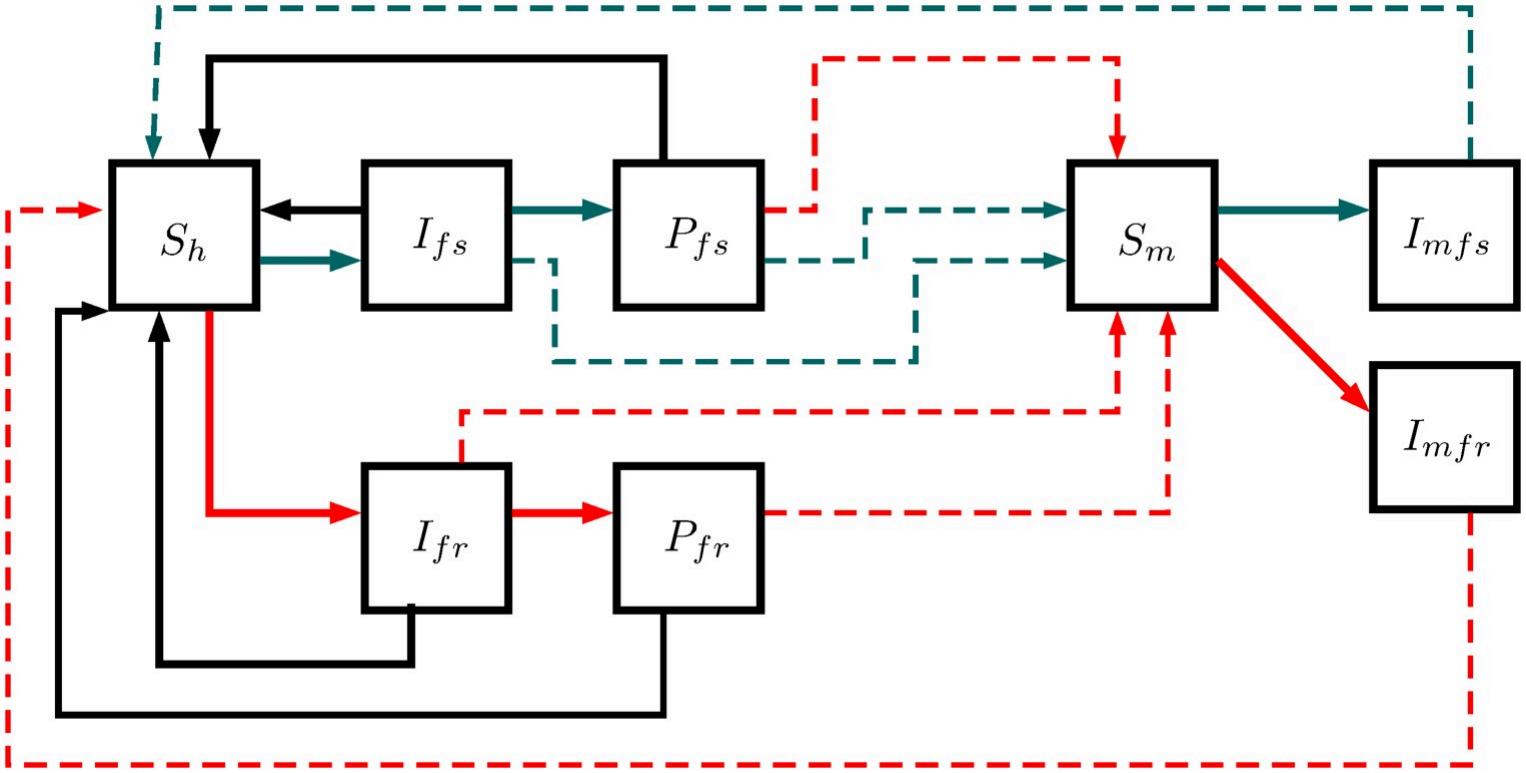
*P. falciparum* model. This structure illustrates the transmission in five human and three mosquito states: susceptible humans *S*_*h*_, infected humans by sensitive strain *I*_*fs*_, post-treatment humans after sensitive infection *P*_*fs*_, infected humans by resistant strain *I*_*fr*_, post-treatment humans after resistant infection *P*_*fr*_, susceptible mosquitoes *S*_*m*_, infected mosquitoes by sensitive strain *I*_*mfs*_, and infected mosquitoes by resistant strain *I*_*mfr*_. Complete lines describe the possible progressions between states, whereas dotted lines describe the parasite transmission between humans and mosquitoes. Red, gray, and black lines display the flows between pairs of states: resistant, sensitive, and recovered states.

This model outlines *P. falciparum* transmission in five human and three mosquito states: susceptible humans *S*_*h*_, infected humans by sensitive strain *I*_*fs*_, post-treatment humans after sensitive infection *P*_*fs*_, infected humans by resistant strain *I*_*fr*_, post-treatment humans after resistant infection *P*_*fr*_, susceptible mosquitoes *S*_*m*_, infected mosquitoes by sensitive strain *I*_*mfs*_, and infected mosquitoes by resistant strain *I*_*mfr*_. Infected and post-treatment humans can infect susceptible mosquitoes, and then, they can become susceptible again (see Fig 1). On the other hand, infected mosquitoes remain in this state until their death due to their short life expectancy. The equations from Eqs 1 to 8 represent the measure per state; Table 1 illustrates model parameters.

**Table 1:** Model parameters.

Parameter	Description	Value
*m*	Mosquitoes per human *N*_*m*_/*N*_*h*_ (dimensionless)	2435/625 [50]
*a*	Biting rate (*day*^−1^)	0.21 [39]
*b*	Transmission probability from an infected mosquito to a susceptible human (dimensionless)	0.5 [51]
*η*	Treatment coverage (dimensionless)	0-1
*σ*_*f*_	Proportion of symptomatic humans infected by *P. falciparum* (dimensionless)	0.9 [52]
*σ*_*v*_	Proportion of symptomatic humans infected by *P. vivax* (dimensionless)	0.33 [53]
*r*_*f*_	Recovery rate of untreated infected-humans by *P. falciparum* (*day*^−1^)	1/287 [50]
*r*_*v*_	Recovery rate of untreated infected-humans by *P. vivax* (*day*^−1^)	1/60 [54]
*γ*_*f*_	Progression rate from infected to post-treatment humans affected by *P. falciparum* (*day*^−1^)	1/2 [50]
*γ*_*v*_	Progression rate from infected to post-treatment humans affected by *P. vivax* (*day*^−1^)	1/9 [36, 55]
*φ*	Proportion of treated humans with primaquine (dimensionless)	0-1
*κ*	Protective period of the treatment (*day*)	see Table 2
*ϵ*_*f*_	Infectious period of post-treatment humans infected by *P. falciparum* (*day*)	see Table 2
*ϵ*_*v*_	Infectious period of post-treatment humans infected by *P. vivax* (*day*)	see Table 2
*α*	Resistance cost (dimensionless)	0-0.6 [24]
*n*	Recurrences produced by the resistant strain (dimensionless)	1
Λ_*m*_	Mosquito birth rate (*day*^−1^)	0.033 [50]
*μ*_*m*_	Mosquito death rate (*day*^−1^). We assumed constant population	0.033
*c*_*a*_	Transmission probability from an asymptomatic human to susceptible mosquito (dimensionless)	0.12 [56]
*c*_*s*_	Transmission probability from an infected-symptomatic human to susceptible mosquito (dimensionless)	0.4 [56]
*ν*	Probability of transmitting a resistant parasite from a post-treatment infected by a sensitive strain (dimensionless)	see Table 2
*ψ*	Hypnozoite relapse rate (*day*^−1^). We assumed tropical relapses	1/60 [57]
*ρ*_*sr*_	Probability of developing sensitive infection by the contact between an infected mosquito by sensitive strain and a human with latent parasites of the resistant strain (dimensionless)	0.5
*ρ*_*rs*_	Probability of developing resistant infection by the contact between an infected mosquito by resistant strain and a human with latent parasites of the sensitive strain (dimensionless)	0.5
*ϕ*_*t*_	Probability of post-treatment human of remaining with latent parasites (dimensionless)	0.21 [58]
*ϕ*_*u*_	Probability of an untreated-infected human of remaining with latent parasites (dimensionless)	0.4-0.9 [59]
*μ*_*vl*_	Clearance rate of latent parasites (hypnozoites) *day*^−1^	1/425 [39]

$$\begin{array}{c} \frac{dS_{h}}{dt}=-mab\frac{I_{mfs}}{N_{m}}S_{h}-\left(1-\alpha \right)mab\frac{I_{mfr}}{N_{m}}S_{h}+\frac{P_{fs}}{\kappa }+\frac{P_{fr}}{\kappa (n+1)}\\ +(1-\eta \sigma_{f})r_{f}I_{fs}+(1-\eta \sigma_{f})r_{f}I_{fr}, \end{array}$$ (1)

$$\begin{array}{c} \frac{dI_{fs}}{dt}=mab\frac{I_{mfs}}{N_{m}}S_{h}-\left(1-\eta \sigma_{f}\right)r_{f}I_{fs}-\eta \sigma_{f}\gamma_{f}I_{fs}, \end{array}$$ (2)

$$\begin{array}{c} \frac{dP_{fs}}{dt}=\eta \sigma_{f}\gamma_{f}I_{fs}-\frac{P_{fs}}{\kappa }, \end{array}$$ (3)

$$\begin{array}{c} \frac{dI_{fr}}{dt}=\left(1-\alpha \right)mab\frac{I_{mfr}}{N_{m}}S_{h}-\left(1-\eta \sigma_{f}\right)r_{f}I_{fr}-\frac{\eta \sigma_{f}\gamma_{f}}{n+1}I_{fr}, \end{array}$$ (4)

$$\begin{array}{c} \frac{dP_{fr}}{dt}=\frac{\eta \sigma_{f}\gamma_{f}}{n+1}I_{fr}-\frac{P_{fr}}{\kappa (n+1)}, \end{array}$$ (5)

$$\begin{array}{c} \frac{dS_{m}}{dt}=\Lambda_{m}N_{m}-\left[ac_{s}\sigma_{f}+ac_{a}\left(1-\sigma_{f}\right)\right]\frac{I_{fs}}{N_{h}}S_{m}-ac_{s}\frac{\epsilon_{f}}{\kappa }\left(1-\varphi \right)\left(1-\nu \right)\frac{P_{fs}}{N_{h}}S_{m}\\ -ac_{s}\frac{{∊}_{f}}{\kappa }\left(1-\alpha \right)\left(1-\varphi \right)\nu \frac{P_{fs}}{N_{h}}S_{m}-\left[ac_{s}\sigma_{f}+ac_{a}\left(1-\sigma_{f}\right)\right]\left(1-\alpha \right)\frac{I_{fr}}{N_{h}}S_{m}\\ -ac_{s}\frac{{∊}_{f}}{\kappa }\left(1-\alpha \right)\left(1-\varphi \right)\frac{P_{fr}}{N_{h}}S_{m}-\mu_{m}S_{m}, \end{array}$$ (6)

$$\begin{array}{c} \frac{dI_{mfs}}{dt}=\left[ac_{s}\sigma_{f}+ac_{a}\left(1-\sigma_{f}\right)\right]\frac{I_{fs}}{N_{h}}S_{m}+ac_{s}\frac{\epsilon_{f}}{\kappa }\left(1-\varphi \right)\left(1-\nu \right)\frac{P_{fs}}{N_{h}}S_{m}-\mu_{m}I_{mfs}, \end{array}$$ (7)

$$\begin{array}{c} \frac{dI_{mfr}}{dt}=\left[ac_{s}\sigma_{f}+ac_{a}\left(1-\sigma_{f}\right)\right]\left(1-\alpha \right)\frac{I_{fr}}{N_{h}}S_{m}+ac_{s}\frac{\epsilon_{f}}{\kappa }\left(1-\alpha \right)\left(1-\varphi \right)\frac{P_{fr}}{N_{h}}S_{m} \end{array}+ac_{s}\frac{{∊}_{f}}{\kappa }\left(1-\alpha \right)\left(1-\phi \right)\nu \frac{P_{fs}}{N_{h}}S_{m}-\mu_{m}I_{mfr},$$ (8)

with

$$\begin{array}{l} N_{h}=S_{h}+I_{fs}+P_{fs}+I_{fr}+P_{fr},\\ N_{m}=S_{m}+I_{mfs}+I_{mfr}. \end{array}$$

### *P. vivax* model

**Fig 2:**
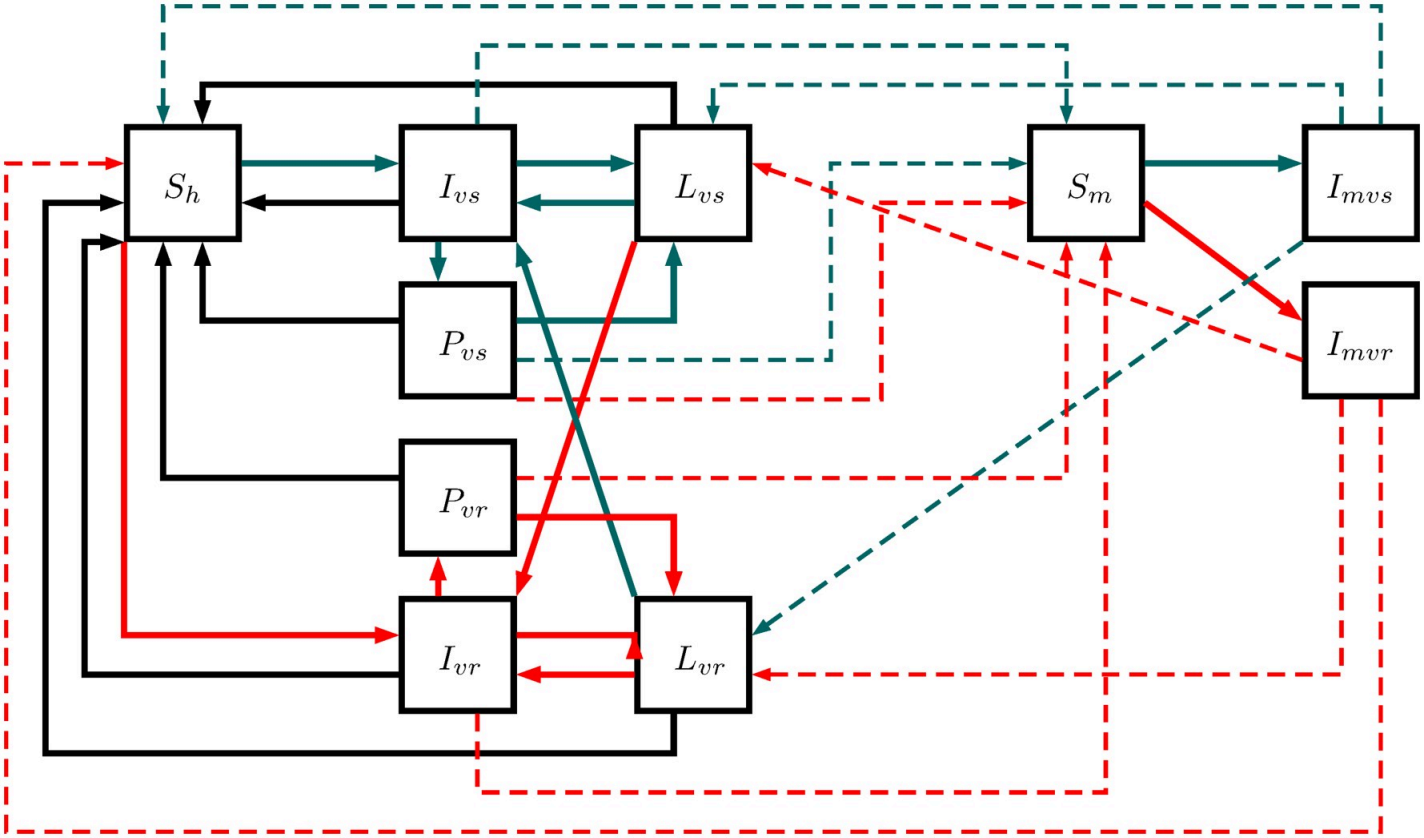
*P. vivax* model. This structure illustrates the transmission in seven human and three mosquito states: susceptible humans *S*_*h*_, infected humans by sensitive strain *I*_*vs*_, humans with latent parasites of sensitive strain *L*_*vs*_, post-treatment humans after sensitive infection *P*_*vs*_, infected humans by resistant strain *I*_*vr*_, humans with latent parasites of resistant strain *L*_*vr*_, post-treatment humans after resistant infection *P*_*vr*_, susceptible mosquitoes *S*_*m*_, infected mosquitoes by sensitive strain *I*_*mvs*_, and infected mosquitoes by resistant strain *I*_*mvr*_. Complete lines reproduce the possible progressions between states while dotted lines reproduce the parasite transmission between humans and mosquitoes. Red lines display the flow of resistant parasites, gray lines display the flow of sensitive parasites, and black lines display the flow without parasites.

This model outlines *P. vivax* transmission in seven human and three mosquito states: susceptible humans *S*_*h*_, infected humans by sensitive strain *I*_*vs*_, humans with latent parasites of sensitive strain *L*_*vs*_, post-treatment humans after sensitive infection *P*_*vs*_, infected humans by resistant strain *I*_*vr*_, humans with latent parasites of resistant strain *L*_*vr*_, post-treatment humans after resistant infection *P*_*vr*_, susceptible mosquitoes *S*_*m*_, infected mosquitoes by sensitive strain *I*_*mvs*_, and infected mosquitoes by resistant strain *I*_*mvr*_. This model reproduces the same transmission interactions of *P. falciparum* model but involves two additional states: *L*_*vs*_ and *L*_*vr*_. These states describe humans with dormant hypnozoites of *P. vivax* that cause relapses after first infection. In fact, *I*_*vs*_, *I*_*vr*_, *P*_*vs*_ and *P*_*vr*_ can remain with latent parasites becoming *L*_*vs*_ or *L*_*vr*_ instead susceptible. Additionally, the model allows new infections in humans with latent parasites as Fig 2 illustrates. The equations are from the Eqs 9 to 18 using the parameters in Table 1.

$$\begin{array}{c} \frac{dS_{h}}{dt}=-mab\frac{I_{mvs}}{N_{m}}S_{h}-mab\left(1-\alpha \right)\frac{I_{mvr}}{N_{m}}S_{h}+\left(1-\eta \sigma_{v}\right)\left(1-\phi_{u}\right)r_{v}\left(I_{vs}+I_{vr}\right) \end{array}+\mu_{vl}\left(L_{vs}+L_{vr}\right)+\frac{\left[1-\phi_{t}\left(1-\varphi \right)\right]}{\kappa }P_{vs}+\frac{\left[1-\phi_{t}\left(1-\varphi \right)\right]}{\kappa (n+1)}P_{vr},$$ (9)

$$\begin{array}{c} \frac{dI_{vs}}{dt}=mab\frac{I_{mvs}}{N_{m}}S_{h}-\left(1-\eta \sigma_{v}\right)r_{v}I_{vs}-\eta \sigma_{v}\gamma_{v}I_{vs}+\psi L_{vs}+mab\rho_{sr}\frac{I_{mvs}}{N_{m}}L_{vr}\\ +mab\frac{I_{mvs}}{N_{m}}L_{vs}+mab\left(1-\rho_{rs}\right)\frac{I_{mvr}}{N_{m}}L_{vs}, \end{array}$$ (10)

$$\begin{array}{c} \frac{dL_{vs}}{dt}=\left(1-\eta \sigma_{v}\right)\phi_{u}r_{v}I_{vs}+\frac{\phi_{t}\left(1-\varphi \right)}{\kappa }P_{vs}-\mu_{vl}L_{vs}-\psi L_{vs}-mab\frac{I_{mvs}}{N_{m}}L_{vs}\\ -mab\left(1-\alpha \right)\frac{I_{mvr}}{N_{m}}L_{vs}, \end{array}$$ (11)

$$\begin{array}{c} \frac{dP_{vs}}{dt}=\eta \sigma_{v}\gamma_{v}I_{vs}-\frac{P_{vs}}{\kappa }, \end{array}$$ (12)

$$\begin{array}{c} \frac{dI_{vr}}{d_{t}}=mab\left(1-\alpha \right)\frac{I_{mvr}}{N_{m}}S_{h}-\left(1-\eta \sigma_{v}\right)r_{v}I_{vr}-\frac{\eta \sigma_{v}\gamma_{v}}{n+1}I_{vr}+\psi L_{vr}+mab\left(1-\alpha \right)\frac{I_{mvr}}{N_{m}}L_{vr}\\ +mab\left(1-\alpha \right)\rho_{rs}\frac{I_{mvr}}{N_{m}}L_{vs}+mab\left(1-\rho_{sr}\right)\frac{I_{mvs}}{N_{m}}L_{vr}, \end{array}$$ (13)

$$\begin{array}{c} \frac{dL_{vr}}{dt}=\left(1-\eta \sigma_{v}\right)\phi_{u}r_{v}I_{vr}+\frac{\phi_{t}\left(1-\varphi \right)}{\kappa (n+1)}P_{vr}-\psi L_{vr}-\mu_{vl}L_{vr}-mab\left(1-\alpha \right)\frac{I_{mvr}}{N_{m}}L_{vr}\\ -mab\frac{I_{mvs}}{N_{m}}L_{vr}, \end{array}$$ (14)

$$\begin{array}{c} \frac{dP_{vr}}{dt}=\frac{\eta \sigma_{v}\gamma_{v}}{n+1}I_{vs}-\frac{P_{vr}}{\kappa (n+1)}, \end{array}$$ (15)

$$\begin{array}{c} \frac{dS_{m}}{dt}=\Lambda_{m}N_{m}-\left[ac_{s}\sigma_{v}+ac_{a}\left(1-\sigma_{v}\right)\right]\frac{I_{vs}}{N_{h}}S_{m}-ac_{s}\frac{\epsilon_{v}}{\kappa }\left(1-\varphi \right)\left(1-\nu \right)\frac{P_{vs}}{N_{h}}S_{m} \end{array}-\left[ac_{s}\sigma_{v}+ac_{a}\left(1-\sigma_{v}\right)\right]\left(1-\alpha \right)\frac{I_{vr}}{N_{h}}S_{m}-ac_{s}\frac{{∊}_{v}}{\kappa }\left(1-\alpha \right)\left(1-\varphi \right)\frac{P_{vr}}{N_{h}}S_{m}-ac_{s}\frac{∊}{\kappa }\left(1-\alpha \right)\left(1-\varphi \right)\nu \frac{P_{vs}}{N_{h}}S_{m}-\mu_{m}S_{m},$$ (16)

$$\begin{array}{c} \frac{dI_{mvs}}{dt}=\left[ac_{s}\sigma_{v}+ac_{a}\left(1-\sigma_{v}\right)\right]\frac{I_{vs}}{N_{h}}S_{m}+ac_{s}\frac{\epsilon_{v}}{\kappa }\left(1-\varphi \right)\left(1-\nu \right)\frac{P_{vs}}{N_{h}}S_{m}-\mu_{m}I_{mvs}, \end{array}$$ (17)

$$\begin{array}{c} \frac{dI_{mvr}}{dt}=\left[ac_{s}\sigma_{v}+ac_{a}\left(1-\sigma_{v}\right)\right]\left(1-\alpha \right)\frac{I_{vr}}{N_{h}}S_{m}+ac_{s}\frac{\epsilon_{v}}{\kappa }\left(1-\alpha \right)\left(1-\varphi \right)\frac{P_{vr}}{N_{h}}S_{m} \end{array}+ac_{s}\frac{{∊}_{v}}{\kappa }\left(1-\alpha \right)\left(1-\varphi \right)\nu \frac{P_{vs}}{N_{h}}S_{m}-\mu_{m}I_{mvr},$$ (18)

with

$$\begin{array}{c} N_{h}=S_{h}+I_{vs}+L_{vs}+P_{vs}+I_{vr}+L_{vr}+P_{vr},\\ N_{m}=S_{m}+I_{mvs}+I_{mvr}. \end{array}$$

### Resistance cost

Resistance cost (*α*) reduces parasite fitness when a mutation occurs and confers resistance on specific treatment [25]. We modeled this cost as a reduction of resistant strains by a multiplicative factor 1 − *α*.

### Asymptomatic infections

We considered asymptomatic infections taking into account the proportion of infected humans with very low malaria parasite density infections. They act as parasite reservoirs, but their transmission rate is lower than the one of symptomatic humans. In the model, the transmission probabilities from asymptomatic and symptomatic individuals to susceptible mosquitoes occur with different probabilities *c*_*a*_ and *c*_*s*_, respectively, considering *c*_*a*_ < *c*_*s*_ [63]. The number of individuals without symptoms is a consequence of the immunological profile in an endemic region due to previous exposition periods. Also, this number varies with parasite [52]. Hence, we considered (1 − *σ*_*f*_) and (1 − *σ*_*v*_) as constant proportion of asymptomatic humans infected by *P. falciparum* and *P. vivax* assuming a long exposition period before treatment.

### Antimalarial treatment

Treatment coverage *η* varies from 0% to 100% of infected humans, adopting a single-treatment regimen. Additionally, the model also permits evaluation of treatment plus primaquine by applying a proportion *φ* of treated humans impacting gametocyte transmission and *P. vivax* hypnozoites.

### Infectious period

Infected without available treatment and asymptomatic humans recover from infection at *r*_*f*_ and *r*_*v*_ rates for *P. falciparum* and *P. vivax*, respectively. Thus, 1/*r*_*f*_ and 1/*r*_*v*_ represent the average infection period, without treatment, for *P. falciparum* and *P. vivax* where 1/*r*_*f*_ > 1/*r*_*v*_ because *P. vivax* model counts only one infection and it can generate a new *P. vivax* relapse when infected human *I*_*v*_ becomes human with latent parasites *L*_*v*_. On the other hand, treated humans advance to post-treatment state at *γ* rate with 1/*γ* as infectious period with 1/*γ*_*v*_ > 1/*γ*_*f*_ because the early development of gametocytes in *P. vivax* triggers longer infectious period before treatment than *P. falciparum* [52, 61]. Resistant parasites provoke recurrences, during treatment, producing more extended infectious periods than infectious periods with sensitive parasites [64]. The mean infectious time for a sensitive strain is 1/*γ* (infectious period), whereas the mean infectious period of a resistant strain is (*n*+ 1)/*γ*, with *n* recurrences. The factor *n*+ 1 captures humans infected by resistant parasite extending their infectious periods when a recurrence occurs.

### Post-treatment period

The post-treatment period engages three dynamics: infectivity, drug half-life, and the emergence of resistant parasites. Parasite clearance of drugs such as chloroquine and artemisinin components affects differently specific parasite forms, i.e., per species [60, 65]. Infectivity depends on the duration of gametocyte presence on blood. We define *ϵ* as the infectious period after treatment; *ϵ* is longer for *P. falciparum* because *P. falciparum* gametocytes have a longer lifespan than *P. vivax* gametocytes (*ϵ*_*f*_ > *ϵ*_*v*_) [36, 65]. Drug half-life *κ* corresponds to the time interval when treatment remains in the blood conferring a protective period [66]. The emergence of resistant parasites occurs by the selection of parasite strains under residual drug concentration, which occurs with probability *ν* of transmitting a resistant parasite from a post-treatment infected by a sensitive strain [23].

### Basic reproduction number

We derived the basic reproduction number adopting the next generation matrix (NGM) approach proposed in [67–69]. The basic reproduction number represents the number of secondary infections generated from an initial primary case in a susceptible population. We assumed constant populations in humans (*N*_*h*_) and mosquitoes (*N*_*m*_) thus *Λ _m_* = *μ* *_m_*. NGM method requires finding the disease–free state (*S*_*h*_ = *N*_*h*_; *S*_*m*_ = *N*_*m*_; the remaining states equal 0) to linearize the equations and building the transmission and transition matrix to derive the basic reproduction numbers [67].

### Simulation

We aim to simulate the spread of drug resistance in *P. falciparum* and *P. vivax* comparing between different treatment-regiments. We contrasted regimens between the adoption of four treatment lines: chloroquine (CQ), chloroquine plus primaquine (CQ+PQ), artemisinin-based combination therapy (ACT) with artemether-lumefantrine (ARLU) and ARLU plus PQ (ACT+PQ). The initial condition is only the presence of the sensitive strain, and Table 2 summarizes the parameters to each treatment regiment. We analyze the system of equations in R using deSolve package [70]. The simulation code is provided in S1 File.

**Table 2:** Treatment parameters.

Treatment regimen	Protective period (*κ*) [60]	Infectious period after treatment (*ϵ*) [36, 60, 61]	Probability of transmitting a resistant parasite from *P*_*fs*_ and *P*_*vs*_ (*ν*) [62]
CQ	30 days	2.1 days (*P. vivax*), 11 days (*P. falciparum*)	10^−12^
CQ+PQ	30 days	2.1 days with (*φ* = 0.95)	10^−12^
ACT (artemether-lumefantrine)	3 days	1.55 days (*P. vivax*), 11 days (*P. falciparum*)	10^−24^
ACT+PQ (artemether-lumefantrine)	3 days	1.55 days (*φ* = 0.95)	10^−24^

### Malaria-transmission settings

We used parameters from the study by Chitnis *et al.* defining low and high transmission settings for a Ross-McDonald structure [50]. We also incorporated the parameter distinction between symptomatic and asymptomatic infectiousness from [56]. *P. falciparum* model showed a valid prevalence (between 0.01 and 0.324) according to the third quartile of 32374 prevalence values reported in the Malaria Atlas Project MAP for 98 countries from 1984 to 2018 [71]. MAP generated a dataset of prevalence values using parasite rate points that came from organizations of health surveys and prevalence studies in literature revision [72]. The *P. vivax* model used the same parameters to make comparable results and also adopted a set parameters to involve hypnozoite relapses [39, 57, 59]. In order to better understand the impact of parameters, we performed a sensitivity analysis to describe the implications of variation in model parameters on the emergence time of resistant strain that represents the moment when resistant-strain prevalence surpasses sensitive-strain prevalence.

### Sensitivity analysis

Finally, we performed a sensitivity analysis of parameter models on the emergence-time of the resistant strain using Latin Hypercube Sampling (LHS) to respond at the uncertainty of estimated values and also to assess the parameter influence [73]. We implemented the analysis in R using deSolve, lhs, and sensitivity packages; the partial correlation coefficients of sensitivity function were calculated with a confidence level of 95% [70, 74, 75]. All reproducibility code is in S1 File.

## Results

### Basic reproduction number

We derived formulae for the basic reproduction number of sensitive and resistant strains in the cases of *P. falciparum* and *P. vivax* models (see from Eqs 19 to 22). These derivations reveal that *R*_0_, as a function of resistant cost *α*, cuts down *R*_0_ values of resistant strains compared to sensitive ones. As expected, recurrences increase the basic reproduction number in both cases. On the other hand, terms associated with latent *P. vivax* parasites reduce the basic reproduction number of both strains in the same proportion.

$$\begin{array}{c} R_{0fs}=\sqrt{\frac{ma^{2}b}{\mu_{m}\left[\left(1-\eta \sigma_{f}\right)r_{f}+\eta \sigma_{f}\gamma_{f}\right]}[c_{s}\sigma_{f}+c_{a}\left(1-\sigma_{f}\right)+\eta \sigma_{f}\gamma_{f}c_{s}\epsilon_{f}\left(1-\nu \right)\left(1-\varphi \right)]} \end{array}$$ (19)

$$\begin{array}{c} R_{0fr}=\left(1-\alpha \right)\sqrt{\frac{ma^{2}b}{\mu_{m}\left[\left(1-\eta \sigma_{f}\right)r_{f}+\frac{\eta \sigma_{f}\gamma_{f}}{n+1}\right]}[c_{s}\sigma_{f}+c_{a}\left(1-\sigma_{f}\right)+\eta \sigma_{f}\gamma_{f}c_{s}\epsilon_{f}\left(1-\varphi \right)]} \end{array}$$ (20)

$$\begin{array}{c} R_{0vs}=\sqrt{\frac{ma^{2}b\left(\psi +\mu_{vl}\right)\left[c_{s}\sigma_{v}+c_{a}\left(1-\sigma_{v}\right)+\eta \sigma_{v}\gamma_{v}c_{s}\epsilon_{v}\left(1-\nu \right)\left(1-\varphi \right)\right]}{\mu_{m}[\left(\psi +\mu_{vl}\right)\left[\left(1-\eta \sigma_{v}\right)r_{v}+\eta \sigma_{v}\gamma_{v}\right]-\psi \left[\phi_{t}\left(1-\varphi \right)\eta \sigma_{v}\gamma_{v}+\phi_{u}\left(1-\eta \sigma_{v}\right)r_{v}\right]]}} \end{array}$$ (21)

$$\begin{array}{c} R_{0vr}=\left(1-\alpha \right)\sqrt{\frac{ma^{2}b\left(\psi +\mu_{vl}\right)\left[c_{s}\sigma_{v}+c_{a}\left(1-\sigma_{v}\right)+\eta \sigma_{v}\gamma_{v}c_{s}\epsilon_{v}\left(1-\varphi \right)\right]}{\mu_{m}[\left(\psi +\mu_{vl}\right)\left[\left(1-\eta \sigma_{v}\right)r_{v}+\frac{\eta \sigma_{v}\gamma_{v}}{n+1}\right]-\psi \left[\frac{\phi_{t}\left(1-\varphi \right)\eta \sigma_{v}\gamma_{v}}{n+1}+\phi_{u}\left(1-\eta \sigma_{v}\right)r_{v}\right]]}} \end{array}$$ (22)

### Simulation

**Fig 3:**
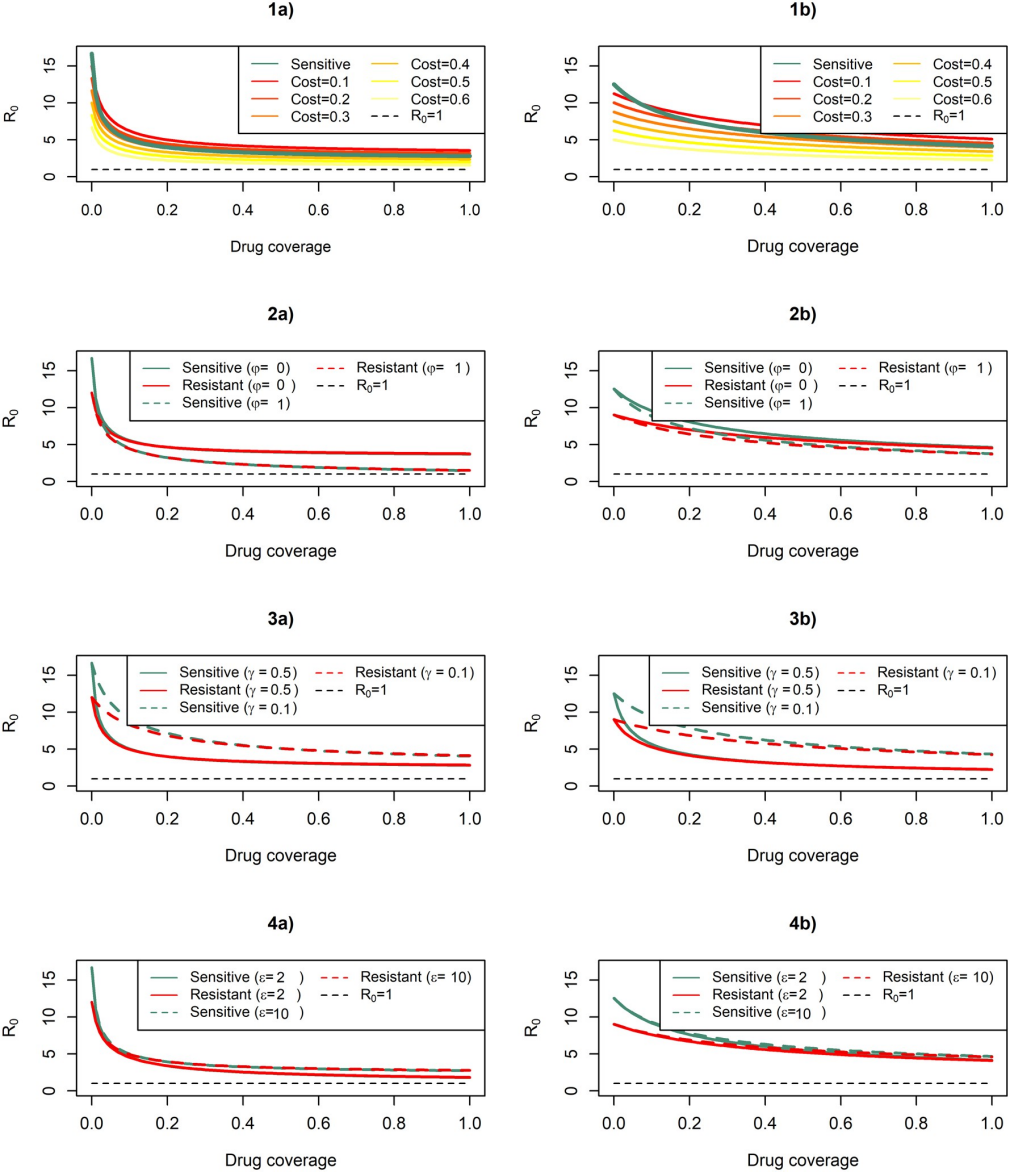
Drug coverage varying the basic reproduction numbers. The figure illustrates *R*_0_ lines for *P. falciparum* (figures a) and *P. vivax* (figures b) models dividing by sensitive and resistant strains. 1(a) and 1(b) display *R*_0_ lines of sensitive and resistant strains with different resistance cost (*α*); 2(a) and 2(b) display *R*_0_ lines using or non-using primaquine (*φ*); 3(a) and 3(b) display *R*_0_ lines at two infectious periods before treatment in days (1/*γ*); 4(a) and 4(b) display *R*_0_ lines at two infectious periods after treatment in days (*ϵ*). *α* = 0.28, *ρ* = 0.5, *ϵ*_*f*_ = 11, *ϵ*_*v*_ = 2.1, *γ*_*f*_ = 1/2 and *γ*_*v*_ = 1/9 when they are fixed; other parameters have values from Table 1.

In numerical simulations, we evaluated the basic reproduction number *R*_0_ by varying cost resistance, treatment plus primaquine, and infectious time before and after treatment (see Fig 3). In general, increases in drug coverage decrease *R*_0_ values of *P. falciparum* at a higher rate than the ones obtained for *P. vivax*. Sensitive *P. vivax* overcomes resistant strains by *R*_0_ values at different resistance cost compared to the switch point for *P. falciparum*. Treatment plus primaquine influences equally *R*_0_ values of sensitives and resistant stains for both species. In general, a longer infectious period before and after treatment increases the reproduction number, but only the longer infectious time before treatment boosted the sensitive *R*_0_ to stay over the resistant *R*_0_. This effect is stronger in *P. vivax* because it has an early transmission before treatment.

**Fig 4:**
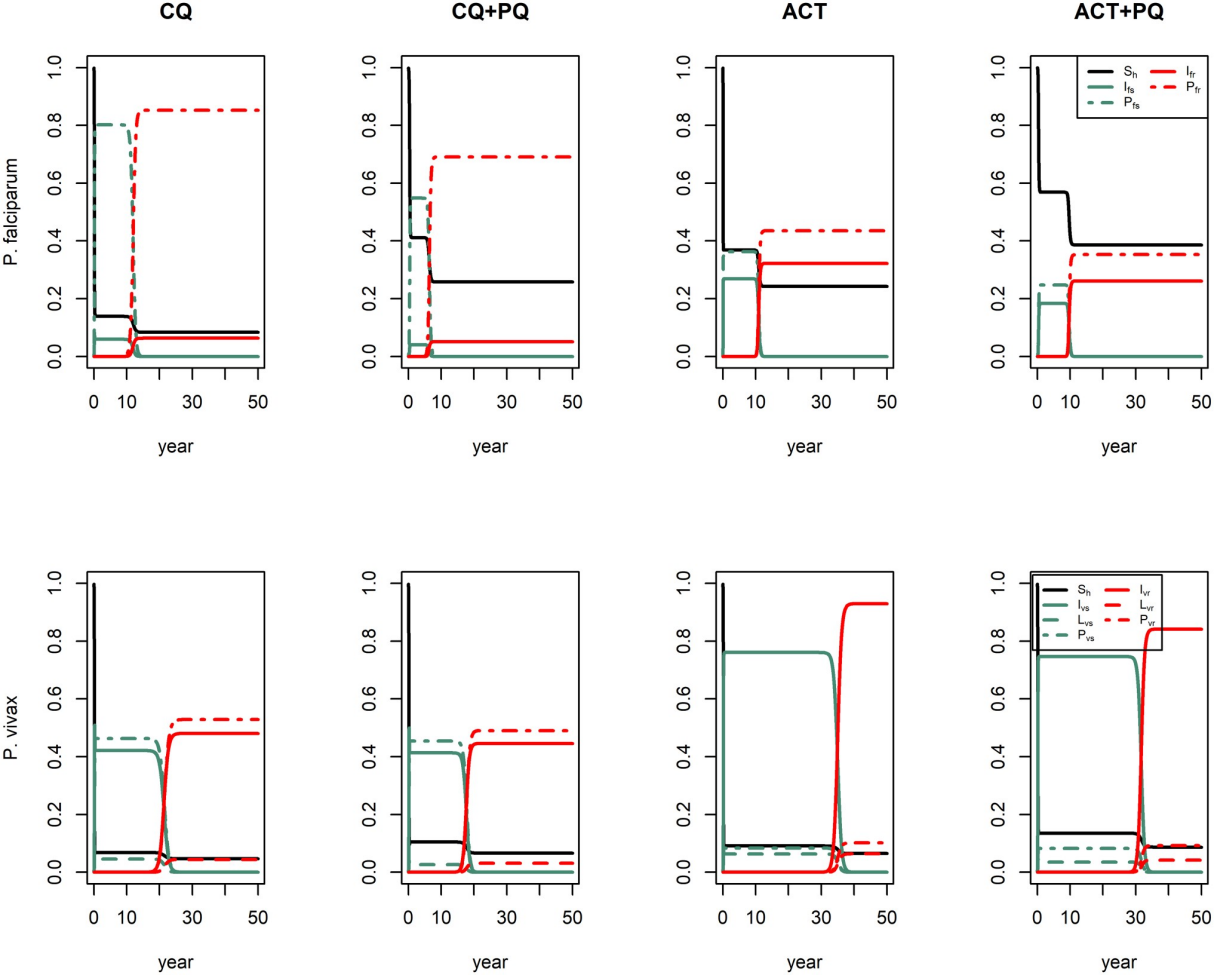
Simulation of treatment regimens. This figure illustrates the implementation of four treatment-regimens: chloroquine CQ (*κ* = 30 days, *ϵ*_*f*_ = 11 days, *ϵ*_*v*_ = 2.1 days, *φ* = 0 and *ν* = 10^−12^), chloroquine plus primaquine CQ+PQ (*κ* = 30 days, *ϵ*_*f*_ = 11 days, *ϵ*_*v*_ = 2.1 days, *φ* = 0.95 and *ν* = 10^−12^), artemisinin-based combination therapy ACT (*κ* = 3 days, *ϵ*_*f*_ = 11 days, *ϵ*_*v*_ = 1.55 days, *φ* = 0 and *ν* = 10^−24^) and artemisinin-based combination therapy plus primaquine ACT+PQ (*κ* = 3 days, *ϵ*_*f*_ = 11 days, *ϵ*_*v*_ = 1.55 days, *φ* = 0.95 and *ν* = 10^−24^). First row shows the simulated regimens in *P. falciparum* model and second row shows the simulated regimens in *P. vivax* model.

Simulations with no regimen produce a proportion of 0.98 for *I*_*vs*_ in *P. falciparum* model and 0.93 and 0.06 for *I*_*vs*_ and *L*_*vs*_, respectively, in *P. vivax* model implying an equilibrium without resistant strain (see Figure A in S2 File). With this initial conditions, we implemented regimen (*η* = 1) obtaining a decrease in all infected proportions in comparison with no regimen but resistant strain emerged in all treatments as Fig 4 illustrates.

Although emergence time of *P. vivax* resistant strain was slower than the one of *P. falciparum* resistant using all regimens, the regimens accomplished a smaller reduction in the proportion of infected humans by *P. vivax*. Treatment with chloroquine (CQ) contributed to a higher proportion of post-treatment humans, especially in the case of *P. falciparum*, and the emergence of resistant *P. vivax* took a time twofold as long as resistant *P. falciparum*. Treatment including primaquine (CQ+PQ) decreased infected and post-treatment humans of *P. falciparum*, and humans with latent parasites of *P. vivax*, but this regimen implied the emergence of resistant parasites in less time.

Regimens with artemisinin-based combination therapy delayed the emergence of resistant *P. vivax* three times as long as resistant *P. falciparum*, but this regimen affected the proportion of infected humans less than chloroquine regimen. Primaquine addition (ACT+PQ) also decreased infected and post-treatment humans of *P. falciparum*, and humans with latent parasites of *P. vivax* though the emergence of resistant parasites remained at a similar time as the ACT without primaquine.

### Sensitivity analysis

**Fig 5:**
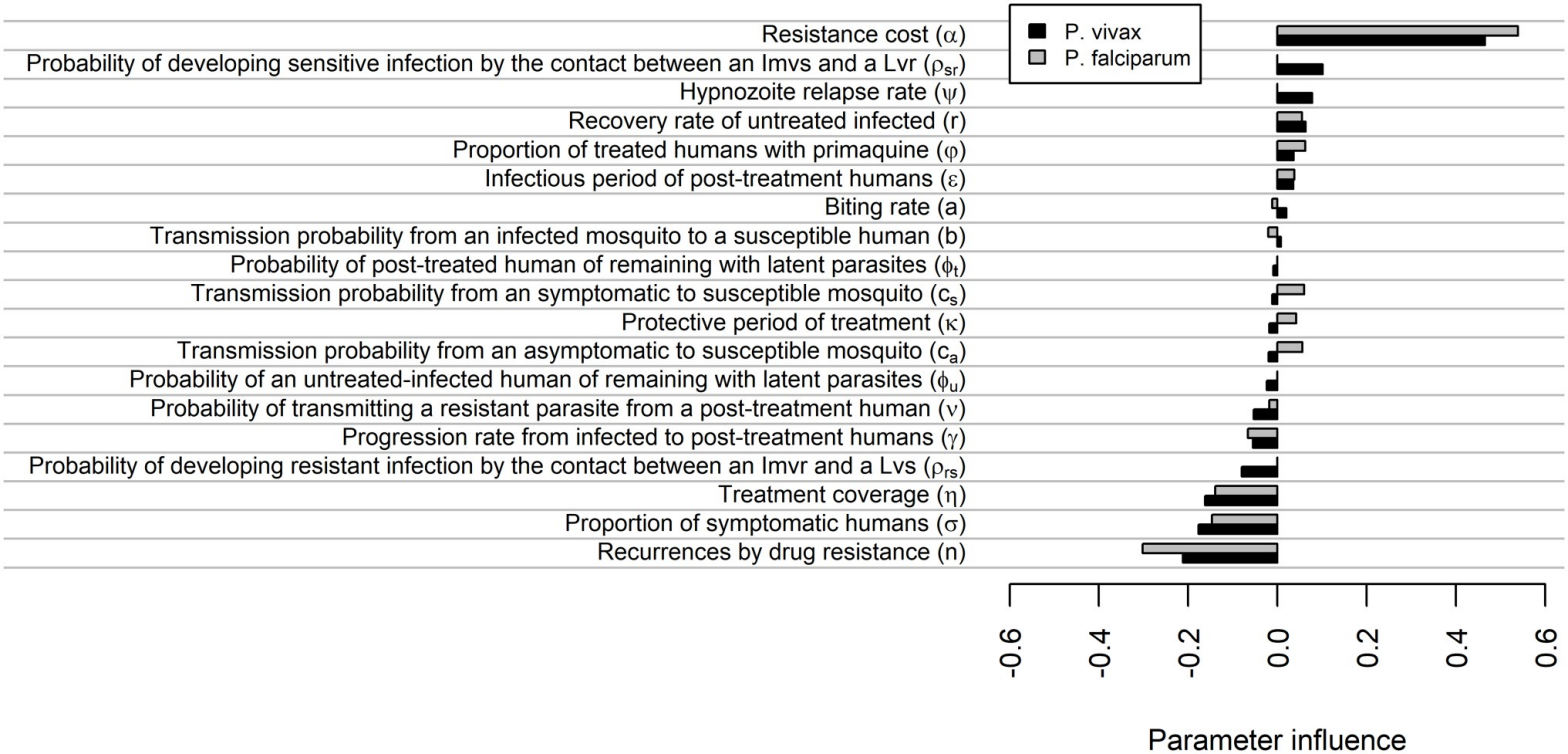
Parameter sensitivity on the emergence-time of the resistant strain. The figure illustrates parameter influence where -1 represents the maximum inverse relation (accelerate drug resistance), 1 represents the maximum proportional relation (delay drug resistance) and 0 represents no relation.

In this model, resistance cost is the most influencing parameter since this parameter delays more the emergence of resistant parasites for either *P. vivax* or *P. falciparum* (see Fig 5). Four parameters were also directly proportional but with a low parameter influence: probability of developing sensitive infection by the contact between *I*_*mvs*_ and a *L*_*vr*_ *ρ*_*sr*_ (only in *P. vivax*), hypnozoite relapse rate *ψ* (only in *P. vivax*), recovery rate of untreated infected *r* and proportion of treated humans with primaquine *φ*. On the other hand, the number of recurrences by drug resistance obtained the most negative influence for both species implying an earlier emergence of the resistant strain. Five parameters also exhibited a negative relationship: the probability of transmitting a resistant parasite from a post-treatment human *ν*, progression rate from infected to post-treatment humans *γ*, probability of developing a resistant infection by the contact between *I*_*mvr*_ and a *L*_*vs*_ *ρ*_*sr*_ (only in *P. vivax*), the proportion of symptomatic humans and treatment coverage. The remaining parameters impacted less, also noting that the transmission probabilities to susceptible mosquito (*c*_*s*_ and *c*_*a*_) and the protective period after treatment *κ* only exhibited a proportional factor for *P. falciparum*.

## Discussion

We found that early transmission before treatment, asymptomatic human, and low effectiveness of drug coverage support the prevalence of sensitive parasites delaying the emergence of resistant *P. vivax*. The reproduction numbers of sensitive *P. vivax* surpassed the reproduction numbers of resistant ones when the infectious period before treatment was greater, and this usually occurs in *P. vivax* transmission by the early development of gametocytes [52]. This effect produces an increase in the number of *P. vivax* infected by a shorter incubation period of parasites as a previous model found [40]. It also implies more difficulties in *P. vivax* elimination and control than *P. falciparum*, illustrating the lowest effectiveness of current treatment regimens against *P. vivax* [45, 76, 77]. Previous models also indicated a lower reduction in *P. vivax* prevalence in same settings than *P. falciparum* [39, 41] but actually, *P. falciparum* prevalence is similar to or greater than *P. vivax* in the same settings suggesting that host acquisition of *P. vivax* immunity would play a role modulating *P. vivax* prevalence [78, 79].

### Implications in treatment policy

Chloroquine (CQ) and Artemisinin-based combination therapy (ACT) with artemether-lumefantrine (ARLU) caused emergence of *P. falciparum* resistance on a similar time scale, whereas ARLU delayed the emergence of *P. vivax* resistance in comparison with CQ. In theory, resistance development to a set of drugs is less likely than a single drug, and this reinforces the improvement of combination therapies [62]. We capture the fact that resistance is less likely for combination therapy by using a smaller *ν* for ACT than CQ (Table 2). Nevertheless, our results showed that fast parasite clearance and shorter protective period of ARLU against *P. falciparum* avoided the transmission of sensitive parasites after-treatment, allowing the emergence period of a resistant parasite as long as the one for CQ despite the lower probability of transmitting resistant parasites with ARLU. CQ resistance in *P. vivax* emerged earlier than ARLU resistance, but CQ achieved a higher reduction in the prevalence of infected humans in overall simulations. These effects indicate that combination therapy evades drug resistance for a long period in *P. vivax*, but its shorter protective period than the one for CQ allows a prevalence increase. This result agrees with the substantial increase in *P. vivax* cases with ACT adoption for all *Plasmodium* species in Papua New Guinea and Indonesia [80]. Hence, combination therapy delays emergence of drug resistance for a long period for *P. vivax* than monotherapy, but it should attach a long protective period to prevent an increase in disease incidence. Indeed, dihydroartemisinin-piperaquine (DPQ) regimen, a combination therapy, is highly recommended for malaria control, since it offers a protective period as well as CQ [60]. Still, extensive use of partner drugs as monotherapy such as piperaquine alone, before combination therapy adoption, would intensify risk of resistance [81]. On the other hand, mixed infections of *P. falciparum*-*P. vivax* produce a premature exposure of *P. vivax* at non-adopted drugs forcing an earlier selective pressure as previous works reported [76, 81, 82]. Our simulation only considered an initial sensitive strain infection without taking into account previous resistance profile and several genotypes, with different drug susceptibility and fitness [83], limiting results to a qualitative validation of regimen adoption in a sensitive population of parasites.

Cessations and changes in drug policy have allowed the emergence of wild-type parasites in endemic zones in Africa and Asia, boosting the possibility of adopting well-known regimens as CQ and sulfadoxine-pyrimethamine [84–87]. Our model structure does not have an inverse mutation from resistant to sensitive strain, but basic reproduction numbers have a comparable structure in terms of parasite competence. If we have a treatment cessation (*η* = 0), *R*_0*fr*_ = (1 − *α*)*R*_0*fs*_ and *R*_0*vr*_ = (1 − *α*)*R*_0*vs*_ entail *R*_0*fs*_ > *R*_0*fr*_ and *R*_0*vs*_ > *R*_0*vr*_, with *α* > 0, generating that sensitive prevalence grows up above resistance prevalence and therefore, reemergence of wild-type strain.

### Impact of treatment plus primaquine

We also tested treatment regimen plus primaquine (PQ) finding that PQ decreased basic reproduction numbers and prevalence of either sensitive or resistant strains. Still, it had a lower contribution to drug-resistance. For *P. falciparum*, PQ helped to avoid transmission after treatment decreasing prevalence using CQ+PQ and ARLU+PQ. Still, CQ resistance occurred slightly earlier because PQ also blocked the transmission of sensitive parasites after treatment in a protective period. PQ decreases *P. vivax* prevalence in humans with latent parasites, but decreases less the prevalence of *P. falciparum*, suggesting that earlier transmission and asymptomatic individuals have a more important role in *P. vivax* transmission than transmission after treatment. This finding agrees with a previous study showing that early transmission in *P. vivax* forced greater reproduction number [45]. This result contrasts with previous studies that found relapses as the most important contributor of *P. vivax* prevalence instead of early transmission [41, 42]. Nevertheless, our results reinforce the adoption of regimens plus a drug to kill hypnozoites and gametocytes, because regimens plus PQ decreased prevalence, when compared to single-drug regimen. In terms of drug resistance, Schneider and Escalante suggested that a drug to kill gametocytes such as PQ could prevent drug resistance [36]. Our model only avoids all transmission of resistant strain with a 100% of PQ regimen which is implausible, given adverse effects of PQ in patients with G6PD deficiency [14, 88]. Simulation results showed an earlier emergence of drug resistance in treatments plus PQ for both *Plasmodium* species. In contrast, sensitivity analysis showed low influence in PQ delaying the emergence of the resistant strain. However, such results are still inconclusive due to potential disturbances in Latin hypercube sampling because *φ* obtained a lower influence. Therefore, our results showed that PQ might not have an essential role in drug resistance over the primary treatment regimen.

### *P. vivax* relapses

Relapses in *P. vivax* constitute the principal challenge in control and elimination programs and incidence increases with more frequent relapses [39, 40, 42]. Our model structure defined relapses through hypnozoite relapse rate (*ψ*) and probabilities of remaining with latent parasites (*φ*_*u*_ and *φ*_*t*_). The formula for *R*_0*v*_ indicated that the hypnozoite relapse rate *ψ* has no important effect on the incidence because this term applies by the same factor in numerator and denominator. The compartmental structure of the model does not capture all the nature of relapse with hypnozoite relapse rate (*ψ*). Nevertheless, probabilities of remaining with latent parasites (*φ*_*u*_ and *φ*_*t*_) help to capture this dynamic, and we found that probabilities *φ*_*u*_ and *φ*_*t*_ increased *R*_0*v*_ and parasite prevalence, connecting with previous studies that noticed greater *R*_0_ with more relapses [39, 41]. Relapses considering drug resistance through sensitivity analysis showed relapse rate as delayer of resistance strain because relapses might benefit the transmission of sensitive parasites before the appearance of the resistant strain. These results suggest that tropical zones support sensitive *P. vivax* more than temperate zones because a single-sensitive infection might cause more than one sensitive infection episode delaying the emergence of resistant strain [41, 89, 90].

### Effect of asymptomatics

The effect of asymptomatic individuals is key because they can support a new transmission way, providing insights into the implication of detection difficulties in *P. vivax*. Basic reproduction numbers expressions illustrated that proportion of symptomatic humans *σ* enhanced the real drug coverage *ση* generating smaller reproduction numbers while asymptomatic proportion (1 − *σ*) increases them. This result agrees with Adapa *et al.* presenting asymptomatics as magnifying the number of cases [45]. Simulation results exhibited a greater prevalence of *P. vivax* (between 40% and 75%) using all regimens in same transmission settings than *P. falciparum* (between 10% and 25%) and larger asymptomatic proportion in *P. vivax* ((1 − *σ*_*v*_)>(1 − *σ*_*f*_)) might explain a part of this greater prevalence. A previous study in the Solomon Islands (low transmission settings) revealed an 82.4% of *P. vivax* prevalence, among which only 2.9% of infected had fever implying a high impact on the transmission of asymptomatic cases linking with model results [91]. In general terms, current results provided some insights into the contributions of asymptomatic reservoirs, but the model structure has limitations. First, models here consider a fixed proportion of asymptomatic, which may vary between localities and time [92]. To perform model robustness, we performed a sensitivity analysis that illustrated *σ* as an accelerator of drug resistance with influence for both *Plasmodium* species. Drug efficacy increases when there is a large proportion of symptomatic humans, boosting the selective force. This result agrees with Kim *et al.* that found lower sensitive-strain fitness with higher drug efficacy [33]. Second, our model structure considers an average decay rate of infected states for both symptomatic and asymptomatic humans at the same levels. The weight of asymptomatic infection might be more significant than expected. In this case, asymptomatic individuals would have a higher impact on disease prevalence and avoiding drug resistance.

### Multiple-genotype infection

The model structure here considers only single genotype infection per infected human, but there is evidence of multiple-genotype infections. Both co-inoculation and superinfection generate multiple genotypes per infected, as reported in endemic zones [93]. Our model does not represent the dynamic of parasite density in blood-stage and the genetic relationship of multiple genotype infections. However, previous studies found that parasite density in blood-stage can limit a subsequent development of new sporozoites [94, 95]. Nkhoma *et al.* found multiple genotype infections with a genetic relationship suggesting a higher probability of single inoculations rather than multiple ones [96]. In general, within-host models can capture better superinfection and co-inoculation because they consider independent parasite stages and parasite densities [22]. Still, we used a compartment model to provide information on the human-population level.

Model structure only allows a superinfection of humans with latent parasites through a new inoculation or activation of present hypnozoites within an activation period. In the case of activation of present hypnozoites, previous studies offered febrile-systematic episodes as activator [35, 90]. Co-activation of hypnozoites of different strains can happen after co-inoculation, but our model does not represent this dynamic because it remains unlikely. Indeed, our model only considers superinfection in a human with latent parasites mediated by *ρ*_*sr*_ and *ρ*_*rs*_. This kind of dynamics captures the superinfection of newly inoculated parasites or activation of latent hypnozoites, either sensitive or resistant. Sensitivity analysis revealed that the parameter given by the probability *ρ*_*sr*_ of an individual with latent parasites of the resistant strain to develop a sensitive infection by inoculation from an infected mosquito with sensitive strain delays drug resistance. By contrast, the probability *ρ*_*rs*_ of a human with latent parasites of sensitive strain to develop resistant infection by mosquito bite with resistant strain accelerates resistance. This result agrees with the study by Klein *et al.* that shows within-host competition as a factor to delay drug resistance [35]. The present models permit to compare risk factors such as hypnozoite-activation or superinfection of humans in latent state and their implications in drug resistance, despite the limited capacity to deal with multiple-genotype infections.

In summary, our results suggest that *P. vivax* has a set of mechanisms to delay drug resistance that implies difficulties in control and elimination programs: earlier transmission, a higher proportion of asymptomatic cases, and relapses. However, programs focused only on symptomatic humans may obtain a weak effect against *P. vivax* prevalence. This strategy is not efficient to block early transmission and asymptomatic reservoirs, despite the strong treatment regimen, longer protective period and an effect killing gametocytes and hypnozoites. Strategies such as mass drug administration impact asymptomatic humans and also avoid earlier transmission, blocking the mechanisms to delay drug resistance of *P. vivax*, but might increase the risk of drug-resistance.

## Supporting information

S1 File Reproducibility code. Code (in R language), parameters and data to reproduce simulation and figures—all figures and model results.
(RMD)

S2 File Document. Document generated from code in S1—Code (in R language), parameters and data to reproduce simulation and figures.
(PDF)
