# Supplementary material for: Early transmission of sensitive strain slows down emergence of drug resistance in Plasmodium vivax
Source: PLoS Comput Biol. 2020 Jun 17;16(6):e1007945. doi: 10.1371/journal.pcbi.1007945 (PMC7363008; doi:10.1371/journal.pcbi.1007945)

Supporting material: R-code figures

Mario J.C. Ayala and Daniel A.M. Villela

Fig 3. Drug coverage varying the basic reproduction numbers

The figure illustrates R0 lines for P. falciparum (figures a) and P. vivax (figures b) models dividing by sensitive and resistant strains. 1(a and 1(b display R0 lines of sensitiveand resistant strains with different resistance cost; 2(a and 2(b display R0 lines using or non-using primaquine; 3(a and 3(b display R0 lines at two infectious periods after treatment in days; 4(a and 4(b display R0 lines at two infectious periods before treatment in days.

```
##########################################################
#                      Functions                         #
#                                                        #
##########################################################
############### Paramaters
# (1) Nm, (2) Nh, (3) a, (4) b, (5) mm, (6) alpha, (7) cs, (8) ca, (9) sigma
# (10)psi, (11) mvl, (12)phit, (13)phiu, (14)varphi, (15)epsilon, (16)nu
# (17)n, (18)gamma, (19)r
#########
######### R0 function
R0<-function(par){
  n<- seq(0,1,0.01)
  R0s<- sqrt((par[1]/par[2]*par[3]^2*par[4]*(par[10]+par[11])*(par[7]*par[9]+par[8]*
                                                               (1-par[9])+n*par[9]*par[18]*par[7]*par
              (par[5]*(((1-n*par[9])*par[19]+n*par[9]*par[18])*(par[10]+par[11])-
                         par[10]*((1-par[14])*n*par[9]*par[18]*par[12]+(1-n*par[9])*par[13]*par[19])))
  R0r<- (1-par[6])*sqrt((par[1]/par[2]*par[3]^2*par[4]*(par[10]+par[11])*(par[7]*
                                                                           par[9]+par[8]*(1-par[9])+n*
                         (par[5]*(((1-n*par[9])*par[19]+n*par[9]*par[18]/(par[17]+1))*(par[10]+par[11]
                                    par[10]*((1-par[14])*n*par[9]*par[18]*par[12]/(par[17]+1)+(1-n*par
                                                par[19]))))
  RF<-data.frame(n,R0s,R0r)
}
########## Plot R0 function
plotR0<-function(especie, # i=1 (P. falciparum), i=2 (P. vivax)
                 parameter, # parameter j={1,...,19}
                 value1, # first value of parameter
                 value2,  # Sencond value of parameter
                 fixed_parameters){
  i<-especie
  j<-parameter
  k<-value1
  l<-value2
  par0<-fixed_parameters
  var<-c(expression(paste("Sensitive (",N[m],"=       ) ")),
         expression(paste("Sensitive (",N[h],"=       ) ")),
         expression(paste("Sensitive (",a,"=       ) ")),
         expression(paste("Sensitive (",b,"=       ) ")),
         expression(paste("Sensitive (",mu[m],"=       )")),
         expression(paste("Sensitive (",alpha,"=       ) ")),
         expression(paste("Sensitive (",c[s],"=       ) ")),
```

```
        expression(paste("Sensitive (",c[a],"=       )")),
        expression(paste("Sensitive (",sigma,"=       ) ")),
        expression(paste("Sensitive (",psi,"=       ) ")),
        expression(paste("Sensitive (",mu[vl],"=       )")),
        expression(paste("Sensitive (",phi[t],"=       ) ")),
        expression(paste("Sensitive (",phi[u],"=       ) ")),
        expression(paste("Sensitive (",varphi,"=  )  ")),
        expression(paste("Sensitive (",epsilon,"=      ) ")),
        expression(paste("Sensitive (",nu,"=       ) ")),
        expression(paste("Sensitive (",n,"=       )")),
        expression(paste("Sensitive (",gamma," =     ) ")),
        expression(paste("Sensitive (",r,"=       ) ")))
  varr<-c(expression(paste("Resistant (",N[m],"=       ) ")),
         expression(paste("Resistant (",N[h],"=       ) ")),
         expression(paste("Resistant (",a,"=       ) ")),
         expression(paste("Resistant (",b,"=       ) ")),
         expression(paste("Resistant (",mu[m],"=       )")),
         expression(paste("Resistant (",alpha,"=       ) ")),
         expression(paste("Resistant (",c[s],"=       ) ")),
         expression(paste("Resistant (",c[a],"=       )")),
         expression(paste("Resistant (",sigma,"=       ) ")),
         expression(paste("Resistant (",psi,"=       ) ")),
         expression(paste("Resistant (",mu[vl],"=       )")),
         expression(paste("Resistant (",phi[t],"=       ) ")),
         expression(paste("Resistant (",phi[u],"=       ) ")),
         expression(paste("Resistant (",varphi,"=  )  ")),
         expression(paste("Resistant (",epsilon,"=     ) ")),
         expression(paste("Resistant (",nu,"=       ) ")),
         expression(paste("Resistant (",n,"=       )")),
         expression(paste("Resistant (",gamma," =     ) ")),
         expression(paste("Resistant (",r,"=       ) ")))
  par<-replace(par0,j,k)
  S1<-R0(par)
  plot(S1$n,S1$R0s,type="l",ylim=c(0,17),col="aquamarine4",lwd=2,xlab="Drug coverage",
       ylab=expression(paste(R[0])),
       cex.lab=1.1,cex.axis=1.1)
  lines(S1$n,S1$R0r,type="l",col="red1",lwd=2)
  par<-replace(par0,j,l)
  S2<-R0(par)
  lines(S2$n,S2$R0s,type="l",ylim=c(0,max(S2$R0s,S2$R0r)),lty=2,col="aquamarine4",lwd=2,
        xlab="Drug coverage",ylab=expression(paste(R[0])))
  lines(S2$n,S2$R0r,type="l",col="red2",lwd=2,lty=2)
  lines(seq(0,1,0.01),seq(0,1,0.01)*0+1,type="l",lty=2,lwd=1,col="black")
  legend("topright", c(var[j],varr[j],var[j],varr[j],
                       expression(paste(R[0],"=1"))),
        col=c("aquamarine4","red","aquamarine4","red","black"), cex = 0.65 ,
        lty=c(1,1,2,2,2),pt.cex = 2)
  text(0.913, 16.65,k,cex = 0.65)
  text(0.913, 15.45,k,cex = 0.65)
  text(0.913, 14.35,l,cex = 0.65)
  text(0.913, 13.15,l,cex = 0.65)
}
#######################################################
```

```
#                                                     #
#                        Figures                      #
#                                                     #
#######################################################
par(mfrow=c(2,4))
######################## Fixed parameters
parf<-c(2435,624,0.21,0.5,0.033,0.28,0.4,0.12,0.9,0,1,0,0,0.5,11,1/10^12,1,1/2,1/287)
# parameter values from 10 to 13 transform R0v in R0f, do not change it.
parv<-c(2435,624,0.21,0.5,0.033,0.28,0.4,0.12,0.33,1/60,1/425,0.29,0.9,0.5,2.1,1/10^12,
        1,1/9,1/60)
##### Figure 1a)
f1a<-R0(parf)
plot(f1a$n,f1a$R0s,type="l",ylim=c(0,17),col="aquamarine4",lwd=2,xlab="Drug coverage",
     ylab=expression(paste(R[0])))
    # cex.lab=1.1,cex.axis=1.1)
title("1a)",lwd=10.58)#,cex.main=1.7)
color<-c("#FF0000FF", "#FF4000FF", "#FF8000FF" ,"#FFBF00FF", "#FFFF00FF", "#FFFF80FF")
for (i in 1:6){
  e=i/10
  parc<-replace(parf,6,e)
  R0r<- R0(parc)
  lines(R0r$n,R0r$R0r,type="l",col=color[i],lwd=2)}
lines(R0r$n,R0r$R0s,type="l",col="aquamarine4",lwd=3)
lines(seq(0,1,0.04),seq(0,1,0.04)*0+1,type="l",lty=2,lwd=1,col="black")
legend("topright", c("Sensitive","Cost=0.1  ","Cost=0.2"
                     ,"Cost=0.3","Cost=0.4",
                     "Cost=0.5","Cost=0.6",
                     expression(paste(R[0],"=1"))),
       col=c("aquamarine4","#FF0000FF", "#FF4000FF", "#FF8000FF" ,"#FFBF00FF", "#FFFF00FF"
             , "#FFFF80FF"
             ,"black"),lty=c(1,1,1,1,1,1,1,2), cex = 0.65 ,pt.cex = 2)
#### Figure 2a
# primaquine, parameter varphi (14)
plotR0(1,14,0,1,parf) # sepecie:falciparum, parameter:varphi, first value:0,
#second value:1, fixed parameters: parf(falciparum)
title("2a)",lwd=10.58)
### Figure 3a
# gamma, parameter (18)
plotR0(1,18,1/2,1/10,parf)
title("3a)",lwd=10.58)
### Figure 4a
# epsilon, parameter (15)
plotR0(1,15,2,10,parf)
title("4a)",lwd=10.58)
##### Figure 1b)
f2b<-R0(parv)
plot(f2b$n,f2b$R0s,type="l",ylim=c(0,17),col="aquamarine4",lwd=2,xlab="Drug coverage",
     ylab=expression(paste(R[0])),
     cex.lab=1.1,cex.axis=1.1)
title("1b)",lwd=10.58)
color<-c("#FF0000FF", "#FF4000FF", "#FF8000FF" ,"#FFBF00FF", "#FFFF00FF", "#FFFF80FF")
for (i in 1:6){
  e=i/10
```

```
  parc<-replace(parv,6,e)
  R0r<- R0(parc)
  lines(R0r$n,R0r$R0r,type="l",col=color[i],lwd=2)}
lines(R0r$n,R0r$R0s,type="l",col="aquamarine4",lwd=3)
lines(seq(0,1,0.04),seq(0,1,0.04)*0+1,type="l",lty=2,lwd=1,col="black")
legend("topright", c("Sensitive","Cost=0.1  ","Cost=0.2"
                     ,"Cost=0.3","Cost=0.4",
                     "Cost=0.5","Cost=0.6",
                     expression(paste(R[0],"=1"))),
       col=c("aquamarine4","#FF0000FF", "#FF4000FF", "#FF8000FF" ,"#FFBF00FF", "#FFFF00FF"
             , "#FFFF80FF"
             ,"black"), cex = 0.65 ,lty=c(1,1,1,1,1,1,1,2),pt.cex = 2)
#### Figure 2b
# primaquine, parameter varphi (14)
plotR0(2,14,0,1,parv) # sepecie:falciparum, parameter:varphi, first value:0,
#second value:1, fixed parameters: parf(falciparum)
title("2b)",lwd=10.58)
### Figure 3b
# gamma, parameter (18)
plotR0(2,18,1/2,1/10,parv)
title("3b)",lwd=10.58)
### Figure 4b
# epsilon, parameter (15)
plotR0(2,15,2,10,parv)
title("4b)",lwd=10.58)
```

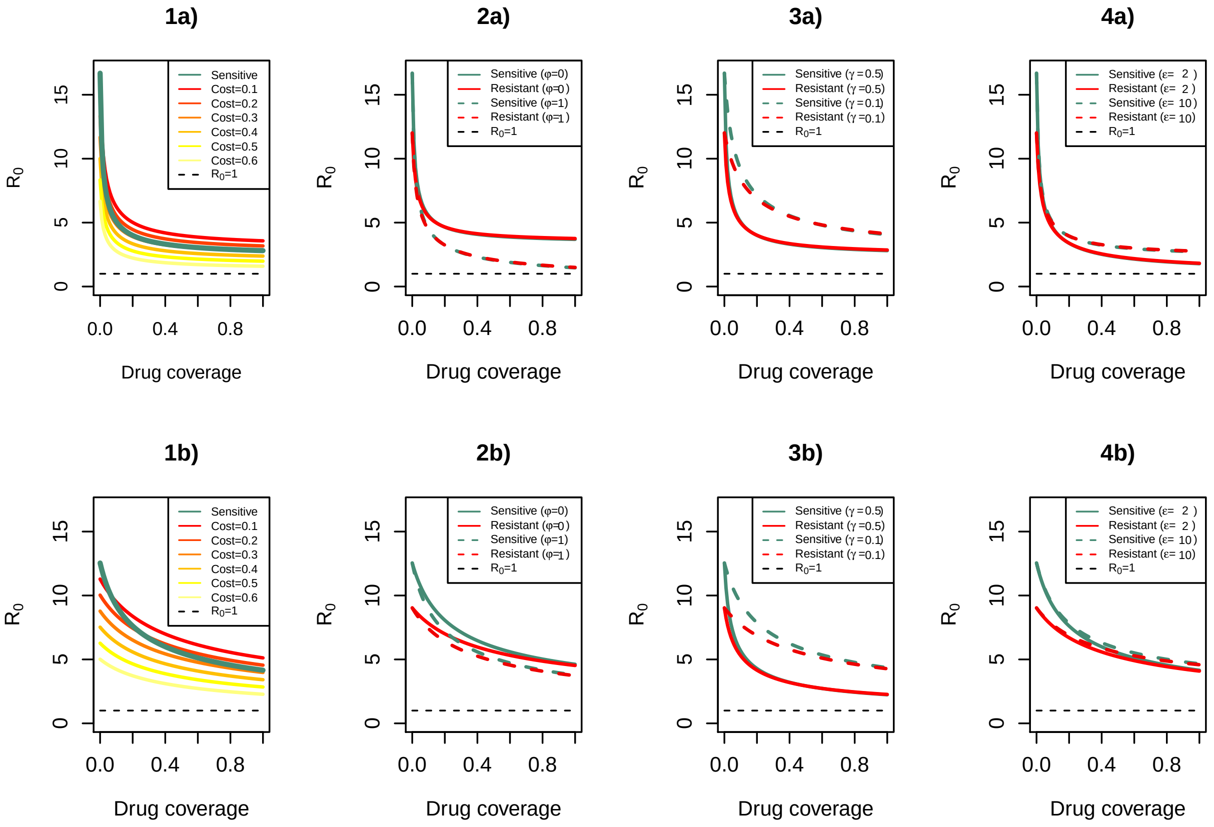

Fig 4. Simulation of treatment regimens

This figure illustrates the implementation of four treatment-regimens:chloroquine (CQ), chloroquine plus primaquine (CQ+PQ), artemisinin combination therapy (ACT) and artemisinincombination therapy plus primaquine (ACT+PQ). First row shows the simulated regimens in P. falciparum model and second row shows the simulated regimens in P. vivax model.

```
### P. falciparum model
falciP <- function(time, y, parms) {
  with(as.list(c(y, parms)),{
    m <- Nm/Nh
    dIs <- m*a*b*Ims/Nm*Sh-(1-n*sf)*rf*Is-n*sf*gf*Is
    dP <- n*sf*gf*Is -P/k
    dIr <- (1-e)*m*a*b*Imr/Nm*Sh-(1-n*sf)*rf*Ir-n*sf*gf/(nr+1)*Ir
    dPr <- n*sf*gf/(nr+1)*Ir-Pr/(k*(nr+1))
    dSh <- -m*a*b*Ims/Nm*Sh+(1-n*sf)*rf*Is-(1-e)*m*a*b*Imr/Nm*Sh+(1-n*sf)*rf*Ir+P/k+
      Pr/(k*(nr+1))
    dIms <- a*cs*sf*Is/Nh*Sm+a*ca*(1-sf)*Is/Nh*Sm+a*cs*ep/k*(1-p)*(1-v)*P/Nh*Sm-mm*Ims
    dImr <-a*cs*sf*(1-e)*Ir/Nh*Sm+a*ca*(1-e)*(1-sf)*Ir/Nh*Sm+
      a*cs*ep/k*(1-e)*(1-p)*Pr/Nh*Sm+a*cs*ep/k*(1-p)*v*(1-e)*P/Nh*Sm-mm*Imr
    dSm <- nm*Nm-a*cs*sf*Is/Nh*Sm-a*ca*(1-sf)*Is/Nh*Sm-a*cs*ep/k*(1-p)*(1-v)*P/Nh*Sm-
      a*cs*sf*(1-e)*Ir/Nh*Sm-a*ca*(1-e)*(1-sf)*Ir/Nh*Sm-
      a*cs*ep/k*(1-e)*(1-p)*Pr/Nh*Sm-a*cs*ep/k*(1-p)*v*(1-e)*P/Nh*Sm-mm*Sm
    list(c(dIs,dP,dIr,dPr,dSh,dIms,dImr,dSm))})
}
### P. vivax model
vivax <- function(time, y, parms) {
  with(as.list(c(y, parms)),{
    m <- Nm/Nh
    dIvs <- m*a*b*Imvs/Nm*Sh-((1-n*sv)*rv+n*sv*gv)*Ivs+psi*Lvs+m*a*b*psr*Imvs/Nm*Lvr+m*a*b*Imvs/Nm*Lvs+
    dLvs <- (1/k)*phit*(1-p)*Pv+(1-n*sv)*rv*phiu*Ivs-mvl*Lvs-psi*Lvs-m*a*b*Imvs/Nm*Lvs-m*a*b*(1-e)*Imvr
    dPv <- n*sv*gv*Ivs-Pv/k
    dIvr <- m*a*b*(1-e)*Imvr/Nm*Sh-((1-n*sv)*rv+n*sv*gv/(nr+1))*Ivr+psi*Lvr+m*a*b*(1-e)*Imvr/Nm*Lvr+m*a
    dLvr <- phiu*(1-n*sv)*rv*Ivr+phit*(1-p)*Pvr/((nr+1)*k)-psi*Lvr-mvl*Lvr-m*a*b*(1-e)*Imvr/Nm*Lvr-m*a*
    dPvr <- n*sv*gv/(nr+1)*Ivr-Pvr/(k*(nr+1))
    dSh <- -m*a*b*Imvs/Nm*Sh-m*a*b*(1-e)*Imvr/Nm*Sh+mvl*(Lvs+Lvr)+(1-n*sv)*(1-phiu)*(rv*Ivs+rv*Ivr)+(1-
    dImvs <- a*cs*sv*Ivs/Nh*Sm+a*ca*(1-sv)*Ivs/Nh*Sm+a*cs*ep/k*(1-v)*(1-p)*Pv/Nh*Sm-mm*Imvs
    dImvr <- a*cs*sv*(1-e)*Ivr/Nh*Sm+a*ca*(1-sv)*(1-e)*Ivr/Nh*Sm+a*cs*ep/k*(1-e)*(1-p)*Pvr/Nh*Sm+a*cs*e
    dSm <- nm*Nm-(a*cs*sv+a*ca*(1-sv))*Ivs/Nh*Sm-(a*cs*sv+a*ca*(1-sv))*(1-e)*Ivr/Nh*Sm-(a*cs*e/k*(1-v)+
    list(c(dIvs,dLvs,dPv,dIvr,dLvr,dPvr,dSh,dImvs,dImvr,dSm))})
}
#### Regimen-simulation function for P. falciparum
regimen_fal <- function(kappa, #Protective period in days
                        epsilon, #Infectious period after treatment in days
                        varphi, # With primaquine = 1, without primaquine = 0
                        therapy, #Monotherapy=1, combinated therapy=2 (two drugs)
                        time # simulation time in years
){
  nu=c(1/10e12,1/10e24)
  time=seq(0,365*time)
  parf <- c(Nm=2435,Nh=624, a=0.21, b=0.5, sf=0.9, n=1, rf=1/287, nr=1, gf=1/2,cs=0.4,
            ca=0.12, mm=0.033,nm=0.033,p=0.95*varphi,k=kappa,ep=epsilon,e=0.1,
            v=nu[therapy])
  y=c(Is=1, P=0, Ir=0,Pr=0,Sh=623,Ims=0,Imr=0,Sm=2435) # initial condition
```

```r
  OP <- ode(y,time,falciP,parf)
  years=OP[,1]/365
  plot(years,OP[,6]/parf[2],xlim=c(min(years),max(years)),ylim=c(0,1),type="l",col="black"
       ,lwd=2,xlab="year",ylab="")
  lines(years,OP[,2]/parf[2],type="l",col="aquamarine4",lwd=2)#Is
  lines(years,OP[,3]/parf[2],type="l",col="aquamarine4", lty=4,lwd=2)#P
  lines(years,OP[,4]/parf[2],type="l",col="red",lwd=2)#Ir
  lines(years,OP[,5]/parf[2],type="l",col="red",lty=4,lwd=2)#Pr
}
#### Regimen-simulation function for P. vivax
regimen_viv <- function(kappa, #Protective period in days
                        epsilon, #Infectious period after treatment in days
                        varphi, # With primaquine = 1, without primaquine = 0
                        therapy, #Monotherapy=1, combinated therapy=2 (two drugs)
                        time # simulation time in years
){
  nu=c(1/10e12,1/10e24)
  time=seq(0,365*time)
  parv <- c(Nm=2435,Nh=624,a=0.21,b=0.5,sv=0.33,n=1,rv=1/60,gv=1/9,cs=0.4,ca=0.12,
            mm=0.033,nm=0.033,p=0.95*varphi,v=nu[therapy],k=kappa,ep=epsilon,e=0.1,psi=1/60,
            psr=0.5,prs=0.5,phit=0.29,phiu=0.9,mvl=1/425,nr=1)
  y=c(Ivs=1, Lvs=0, Pv=0, Ivr=0, Lvr=0, Pvr=0, Sh=622, Imvs=0, Imvr=0, Sm=2435)
  OP <- ode(y,time,vivax,parv,method = "ode2")
  years=OP[,1]/365
  plot(years,OP[,8]/parv[2],xlim=c(min(years),max(years)),ylim=c(0,1),type="l",col="black",lwd=2,
       xlab="year",ylab="")
  lines(years,OP[,2]/parv[2],type="l",col="aquamarine4",lwd=2)#Ivs
  lines(years,OP[,3]/parv[2],type="l",col="aquamarine4",lwd=2,lty=2)#Lvs
  lines(years,OP[,4]/parv[2],type="l",col="aquamarine4",lwd=2,lty=4)#Pvs
  lines(years,OP[,5]/parv[2],type="l",col="red",lwd=2)#Ivr
  lines(years,OP[,6]/parv[2],type="l",col="red",lwd=2,lty=2)#Lvr
  lines(years,OP[,7]/parv[2],type="l",col="red",lwd=2,lty=4)#Pvr
}
######### Regimen simulations and figures ##############
par(mfrow=c(2,4))
###### CQ for P. falciparum
regimen_fal(30,11,0,1,50)
title(main="CQ",ylab="P. falciparum",lwd=10.58)
##### CQ+PQ for P. falciparum
regimen_fal(30,11,1,1,50)
title(main="CQ+PQ",lwd=10.58)
###### ACT for P. falciparum
regimen_fal(3,11,0,2,50)
title(main="ACT",lwd=10.58)
###### ACT+PQ for P. falciparum
regimen_fal(3,11,1,2,50)
title(main="ACT+PQ",lwd=10.58)
legend("topright", legend=c(TeX('$S_h$'),TeX('$I_{fs}$'),
                            TeX('$P_{fs}$'),TeX('$I_{fr}$'),TeX('$P_{fr}$')),
       col=c("black","aquamarine4","aquamarine4","red","red"), lty=c(1,1,4,1,4), cex=0.7,
       lwd=2,ncol=2)
###### CQ for P. vivax
regimen_viv(30,2.1,0,1,50)
```

```
title(ylab="P. vivax",lwd=10.58)
##### CQ+PQ for P. vivax
regimen_viv(30,2.1,1,1,50)
###### ACT for P. vivax
regimen_viv(3,1.55,0,2,50)
title(cex.lab=1.7)
###### ACT+PQ for P. vivax
#par(mar=c(5, 4, 3,3), xpd=TRUE)
re<-regimen_viv(3,1.55,1,2,50)
legend("topleft",legend=c(TeX('$S_h$'),TeX('$I_{vs}$'),TeX('$L_{vs}$'),TeX('$P_{vs$}')
                          ,TeX('$I_{vr}$'),TeX('$L_{vr}$'),TeX('$P_{vr$}')),
       col=c("black","aquamarine4","aquamarine4","aquamarine4","red","red","red"),
       lty=c(1,1,2,4,1,2,4), cex=0.68,lwd=2,ncol=2,bg="transparent",inset=.01)
```

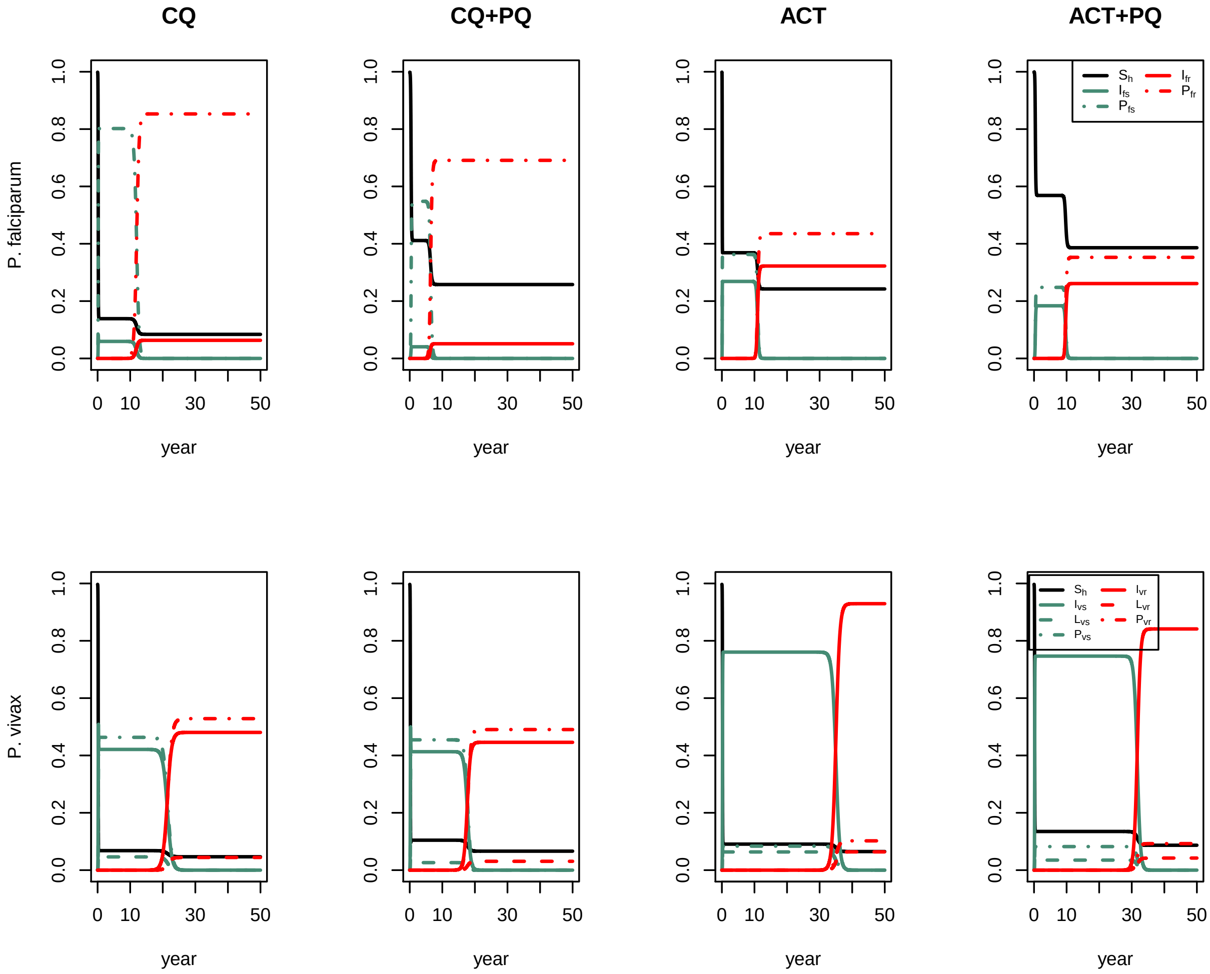

Fig 5. Parameter sensitivity on the emergence-time of the resistant strain

The figure illustrates parameterinfluence where -1 represents the maximum inverse relation (accelerate drug resistance), 1 represents the maximumproportional relation (delay drug resistance) and 0 represents no relation.

```r
res_viv<-c(0.019585114,0.008129122,-0.176634662,0.063102445,-0.211633090
           ,-0.055122506,-0.011876919,-0.020322021,0.035809343,-0.053049253
           ,-0.018142337,0.035521216,0.464934960,0.077976719,0.101080519
           ,-0.080115639,-0.009622503,-0.024275347,-0.162420022)
res_fal<-c(-0.01139271, -0.02097551, -0.14643732,  0.05480114, -0.30216895,
           -0.06653930,  0.06016104,  0.05533678,  0.06228226, -0.01864692,
           0.04187478,  0.03805082,  0.53898674,0,0,0,0,0,-0.13969531)
novo_sen<-rbind(res_viv,res_fal)
para=c("Biting rate",
       "Transmission probability from an infected mosquito to a susceptible human",
       "Proportion of symptomatic humans",
       "Recovery rate of untreated infected",
       "Recurrences by drug resistance",
       "Progression rate from infected to post-treatment humans",
       "Transmission probability from an symptomatic to susceptible mosquito",
       "Transmission probability from an asymptomatic to susceptible mosquito",
       "Proportion of treated humans with primaquine",
       "Probability of transmitting a resistant parasite from a post-treatment human",
       "Protective period of the treatment",
       "Infectious period of post-treatment humans",
       "Resistance cost",
       "Hypnozoite relapse rate",
       "Probability of developing sensitive infection by the contact between an Imvs and a
       Lvr",
       "Probability of developing resistant infection by the contact between an Imvr and a
       Lvs",
       "Probability of post-treated human of remaining with latent parasites",
       "Probability of an untreated-infected human of remaining with latent parasites",
       "Treatment coverage")
data_order<-rbind(para,novo_sen)
trek<-data.frame(t(data_order))
names(trek)<-c("X1","X2","X3")
trek<-trek[order(trek$X2),]
fin<-t(rbind(trek[11:1,],trek[12:19,]))
viv1<-as.numeric(fin[2,])[1:19]
fal1<-as.numeric(fin[3,])[1:19]
varT<-rbind(viv1,fal1)
# automatic names (without math expression)
#nam<-as.character(fin[1,])[1:19]
# Fixed math expression
nam<-c(expression(paste("Resistance cost (",alpha,")")),
       expression(paste("Probability of developing sensitive infection by the contact between an Imvs ar
       expression(paste("Hypnozoite relapse rate (",psi,")")),
       "Recovery rate of untreated infected (r)",
       expression(paste("Proportion of treated humans with primaquine (",varphi,")")),
       expression(paste("Infectious period of post-treatment humans (",epsilon,")")),
       "Biting rate (a)",
       "Transmission probability from an infected mosquito to a susceptible human (b)",
       expression(paste("Probability of post-treated human of remaining with latent parasites (",phi[t]
       expression(paste("Transmission probability from an symptomatic to susceptible mosquito (",c[s],"
       expression(paste("Protective period of treatment (",kappa,")")),
       expression(paste("Transmission probability from an asymptomatic to susceptible mosquito (",c[a],
       expression(paste("Probability of an untreated-infected human of remaining with latent parasites
```

```
        expression(paste("Probability of transmitting a resistant parasite from a post-treatment human (
        expression(paste("Progression rate from infected to post-treatment humans (",gamma,")")),
        expression(paste("Probability of developing resistant infection by the contact between an Imvr an
        expression(paste("Treatment coverage (",eta,")")),
        expression(paste("Proportion of symptomatic humans (",sigma,")")),
        "Recurrences by drug resistance (n)"
)
par(xpd=NA,oma=c(0,17,0,0))
barplot(varT,
        xlab="",ylab="",xlim=c(-0.6,0.6),
        names.arg=nam[19:1],cex.axis=0.7,
        col=c("black","gray"),
        beside=TRUE,horiz = TRUE,las=2,cex.names=0.6)
abline(h=seq(0.5,60,by=3), col="gray", lty=1)
abline(v=seq(0.65,1,by=0.001), col="white", lty=1,lwd=3)
abline(h=seq(58,80,by=0.01), col="white", lty=1,lwd=3)
abline(h=seq(58,80,by=0.01), col="white", lty=1,lwd=3)
legend("topleft",legend=c("P. vivax","P. falciparum"),fill=c("black","gray"), cex=0.6)
title(xlab="Parameter influence",cex.lab=0.8)
```

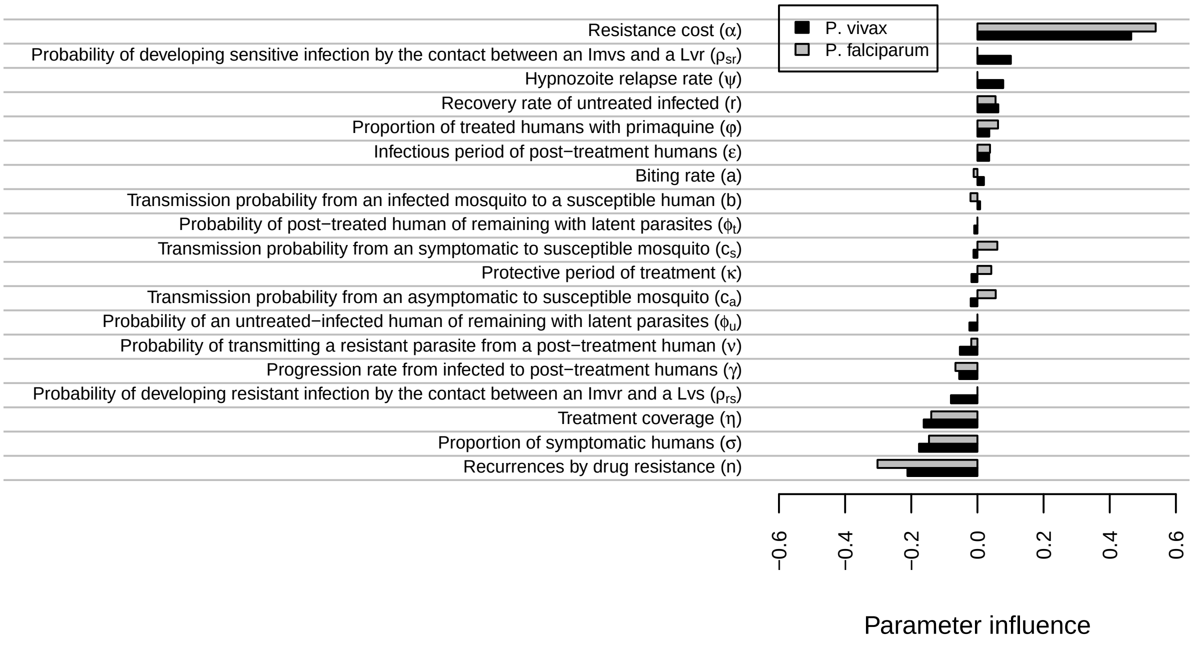

We performed the coeficients of parameter influence through next code:

P. vivax model:

Initially, we put the function of model equations:

```
Vivax <- function(t, x, params){
  Ivs <- x[1]
  Lvs <- x[2]
```

```
  Pv <- x[3]
  Ivr <- x[4]
  Lvr <- x[5]
  Pvr <- x[6]
  Sh <- x[7]
  Imvs <- x[8]
  Imvr <- x[9]
  Sm <- x[10]
  Nm<-2435
  Nh<-624
  mm<-0.033
  nm<-0.033
  nh<-0
  mh<-0
  mvl=1/425
  m <- Nm/Nh
  dIvs <- m*params$a*params$b*Imvs/Nm*Sh-((1-params$n*params$sv)*params$rv+
params$n*params$sv*params$gv)*Ivs+params$psi*Lvs-mh*Ivs+
    m*params$a*params$b*params$psr*Imvs/Nm*Lvr+
    m*params$a*params$b*Imvs/Nm*Lvs+m*params$a*params$b*(1-params$prs)*Imvr/Nm*Lvs
  dLvs <- (1/params$k)*params$phit*(1-params$p)*Pv+(1-params$n*params$sv)*params$rv*
    params$phiu*Ivs-
mvl*Lvs-params$psi*Lvs-mh*Lvs-m*params$a*params$b*Imvs/Nm*Lvs-
    m*params$a*params$b*(1-params$e)*Imvr/Nm*Lvs
  dPv <- params$n*params$sv*params$gv*Ivs-Pv/params$k-mh*Pv
  dIvr <- m*params$a*params$b*(1-params$e)*Imvr/Nm*Sh-
    ((1-params$n*params$sv)*params$rv+params$n*params$sv*params$gv/(params$nr+1))*Ivr+
params$psi*Lvr-mh*Ivr+m*params$a*params$b*(1-params$e)*Imvr/Nm*Lvr+
m*params$a*params$b*(1-params$e)*params$prs*Imvr/Nm*Lvs+
m*params$a*params$b*(1-params$psr)*Imvs/Nm*Lvr
  dLvr <- params$phiu*(1-params$n*params$sv)*params$rv*Ivr+
   params$phit*(1-params$p)*Pvr/((params$nr+1)*params$k)-params$psi*Lvr-mvl*Lvr-mh* Lvr-
   m*params$a*params$b*(1-params$e)*Imvr/Nm*Lvr-m*params$a*params$b*Imvs/Nm*Lvr
  dPvr <- params$n*params$sv*params$gv/(params$nr+1)*Ivr-Pvr/(params$k*(params$nr+1))-
  mh*Pv
dSh <- nh*Nh-m*params$a*params$b*Imvs/Nm*Sh-m*params$a*params$b*(1-params$e)*Imvr/Nm*Sh+
mvl*(Lvs+Lvr)+(1-params$n*params$sv)*(1-params$phiu)*(params$rv*Ivs+params$rv*Ivr)+
(1-params$phit*(1-params$p))/params$k*Pv+(1-params$phit*(1-params$p))/
  (params$k*(params$nr+1))*Pvr-mh*Sh
dImvs <- params$a*params$cs*params$sv*Ivs/Nh*Sm+params$a*params$ca*(1-params$sv)*Ivs/Nh*Sm+
  params$a*params$cs*params$ep/params$k*(1-params$v)*(1-params$p)*Pv/Nh*Sm-mm*Imvs
  dImvr <- params$a*params$cs*params$sv*(1-params$e)*Ivr/Nh*Sm+
  params$a*params$ca*(1-params$sv)*(1-params$e)*Ivr/Nh*Sm+
  params$a*params$cs*params$ep/params$k*(1-params$e)*(1-params$p)*Pvr/Nh*Sm+
  params$a*params$cs*params$ep/params$k*(1-params$e)*(1-params$p)*params$v*Pv/Nh*Sm-
  mm*Imvr
  dSm <- nm*Nm-
  (params$a*params$cs*params$sv+params$a*params$ca*(1-params$sv))*Ivs/Nh*Sm-
  (params$a*params$cs*params$sv+params$a*params$ca*(1-params$sv))*(1-params$e)*Ivr/Nh*Sm-
  (params$a*params$cs*params$e/params$k*(1-params$v)+params$a*params$cs*params$ep/params$k*
(1-params$e)*params$v)*(1-params$p)*Pv/Nh*Sm-
  params$a*params$cs*params$ep/params$k*(1-params$e)*(1-params$p)*Pvr/Nh*Sm-mm*Sm
  list(c(dIvs,dLvs,dPv,dIvr,dLvr,dPvr,dSh,dImvs,dImvr,dSm))
```

```
}
require(deSolve)
times <- seq(0,365*50, by=1) #solve for 50 years
```

Then, we made the latin hypercube sampling of 500 point per parameter. We established a parameter range using the sources from main text (table 1, model parameters).

```
########### Latin hypercube sampling ###########
require(lhs) #add the lhs library
h <- 500 #choose number of points
lhs<-maximinLHS(h,18) #simulate ### 18parameters ###
##### Parameter range #####
#a=0.21
a.min<-0.1
a.max<-1
#b=0.5
b.min<-0.45
b.max<-0.65
#sv=0.33
sv.min<-0
sv.max<-1
# rv=1/60
rv.min<-0.0014
rv.max<-0.017
#nr=1
nr.min<-0
nr.max<-2
#gf=1/2
gv.min<-1/15
gv.max<-1/6
#cs=0.4
cs.min<-0.16
cs.max<-0.4
#ca=0.12
ca.min<-0.05
ca.max<-0.17
#p=0.95*0
p.min<-0
p.max<-0.95
#v=1/10e12
v.min<-1/10e24
v.max<-1/10e12
#k=30
k.min<-3
k.max<-30
#ep=11
ep.min<-1
ep.max<-30
#e=0.1
e.min<-0.05
e.max<-1
#psi<-1/60
psi.min <- 1/365
psi.max <- 1/30
```

```
#psr=0.5
psr.min <- 0
psr.max <- 1
#prs=0.5
prs.min <- 0
prs.max <- 1
# phit <- 0.29
phit.min <- 0
phit.max <- 1
# phiu <- 0.9
phiu.min <- 0
phiu.max <- 1
##### latin hypercube sample
params.set <- cbind(
  a = lhs[,1]*(a.max-a.min)+a.min,
  b = lhs[,2]*(b.max-b.min)+b.min,
  sv= lhs[,3]*(sv.max-sv.min)+sv.min,
  rv= lhs[,4]*(rv.max-rv.min)+rv.min,
  nr= lhs[,5]*(nr.max-nr.min)+nr.min,
  gv= lhs[,6]*(gv.max-gv.min)+gv.min,
  cs= lhs[,7]*(cs.max-cs.min)+cs.min,
  ca= lhs[,8]*(ca.max-ca.min)+ca.min,
  p = lhs[,9]*(p.max-p.min)+p.min,
  v = lhs[,10]*(v.max-v.min)+v.min,
  k = lhs[,11]*(k.max-k.min)+k.min,
  ep= lhs[,12]*(ep.max-ep.min)+ep.min,
  e = lhs[,13]*(e.max-e.min)+e.min,
  psi=lhs[,14]*(psi.max-psi.min)+psi.min,
  psr=lhs[,15]*(psr.max-psr.min)+psr.min,
  prs=lhs[,16]*(prs.max-prs.min)+prs.min,
  phit=lhs[,17]*(phit.max-phit.min)+phit.min,
  phiu=lhs[,18]*(phiu.max-phiu.min)+phiu.min
)
####### treatment coverage n ####
l <- 40
##########  simulating points ########
h2 <-500
```

We obtained the emergence time (when the prevalence of resistant strain overcomes the prevalence of sensitive strain) per sampling:

```
### 18 varaibles + n + emergence = 20 ###
j <- 1
data <- data.frame(matrix(rep(NA,l*h2*20),nrow=l*h2))
for(i in 1:h2){
  for (n in seq(0,1,length=l)){
    xstart<- c(Ivs=1,Lvs=0,Pv=0,Ivr=0,Lvr=0,Pvr=0,Sh=622,Imvs=0,Imvr=0,Sm=2435)
    params <- as.list(c(params.set[i,],n=n))
    out <- as.data.frame(lsoda(xstart, times, Vivax, params))
    emergence<-(365*50-sum(as.numeric(out$Ivr>out$Ivs)))/365
    data[j,1:19] <- params
    data[j,20] <- emergence
    j <- j+1
  }
```

```
}
names(data) <- c(names(params),'Emergence')
```

Finally, we obtained the parameter influence using the emergence time from "data" matrix

```
#### Sensitivity ####
require(sensitivity)
library(latex2exp)
sad <- pcc(data[,1:19], data[,20], nboot = 100, rank=TRUE)
```

We implemented the same process with the P. falciparum model:

```
falciP <- function(t, x, params){
Is <- x[1]
P <- x[2]
Ir <- x[3]
Pr <- x[4]
Sh <- x[5]
Ims <- x[6]
Imr <- x[7]
Sm <- x[8]
Nm<-2435
Nh<-624
mm<-0.033
nm<-0.033
nh<-0
mh<-0
m <- Nm/Nh
dIs <- m*params$a*params$b*Ims/Nm*Sh-(1-params$n*params$sf)*params$rf*Is-params$n
*params$sf*params$gf*Is-mh*Is
dP <- params$n*params$sf*params$gf*Is -P/params$k-mh*P
dIr <- (1-params$e)*m*params$a*params$b*Imr/Nm*Sh-(1-params$n*params$sf)*params$rf*
  Ir-params$n*params$sf*params$gf/(params$nr+1)*Ir-mh*Ir
dPr <- params$n*params$sf*params$gf/(params$nr+1)*Ir-Pr/(params$k*(params$nr+1))-mh*Pr
dSh <- -m*params$a*params$b*Ims/Nm*Sh+(1-params$n*params$sf)*params$rf*Is-(1-params$e)*
  m*params$a*params$b*Imr/Nm*Sh+(1-params$n*params$sf)*params$rf*Ir+P/params$k+
  Pr/(params$k*(params$nr+1))+nh*Nh-mh*Sh
dIms <- params$a*params$cs*params$sf*Is/Nh*Sm+params$a*params$ca*(1-params$sf)*
  Is/Nh*Sm+params$a*params$cs*params$ep/params$k*(1-params$p)*(1-params$v)*P/Nh*Sm-mm*Ims
dImr <- params$a*params$cs*params$sf*(1-params$e)*Ir/Nh*Sm+params$a*params$ca*
  (1-params$e)*(1-params$sf)*Ir/Nh*Sm+params$a*params$cs*params$ep/params$k*
  (1-params$e)*(1-params$p)*Pr/Nh*Sm+params$a*params$cs*params$ep/params$k*
  (1-params$p)*params$v*(1-params$e)*P/Nh*Sm-mm*Imr
dSm <- nm*Nm-params$a*params$cs*params$sf*Is/Nh*Sm-params$a*params$ca*(1-params$sf)*
  Is/Nh*Sm-params$a*params$cs*params$ep/params$k*(1-params$p)*(1-params$v)*P/Nh*Sm-
  params$a*params$cs*params$sf*(1-params$e)*Ir/Nh*Sm-params$a*params$ca*(1-params$e)*
  (1-params$sf)*Ir/Nh*Sm-params$a*params$cs*params$ep/params$k*(1-params$e)*(1-params$p)*
  Pr/Nh*Sm-params$a*params$cs*params$ep/params$k*(1-params$p)*params$v*(1-params$e)*
  P/Nh*Sm-mm*Sm
list(c(dIs,dP,dIr,dPr,dSh,dIms,dImr,dSm))
}
require(deSolve)
times <- seq(0,365*50, by=1) #solve for 50 years
########### Latin hypercube sampling ###########
require(lhs) #add the lhs library
```

```r
h <- 500 #choose number of points
lhs<-maximinLHS(h,13) #simulate ### 13 parameters ###
##### Parameter range #####
#a=0.21
a.min<-0.
a.max<-1
#b=0.5
b.min<-0.45
b.max<-0.65
#sf=0.9
sf.min<-0
sf.max<-1
# rf=1/287
rf.min<-0.0014
rf.max<-0.017
#nr=1
nr.min<-0
nr.max<-2
#gf=1/2
gf.min<-1/15
gf.max<-1
#cs=0.4
cs.min<-0.16
cs.max<-0.4
#ca=0.12
ca.min<-0.05
ca.max<-0.17
#p=0.95*0
p.min<-0
p.max<-0.95
#v=1/10e12
v.min<-1/10e24
v.max<-1/10e12
#k=30
k.min<-3
k.max<-30
#ep=11
ep.min<-1
ep.max<-30
#e=0.1
e.min<-0.05
e.max<-1
##### latin hypercube sample
params.set <- cbind(
a = lhs[,1]*(a.max-a.min)+a.min,
b = lhs[,2]*(b.max-b.min)+b.min,
sf= lhs[,3]*(sf.max-sf.min)+sf.min,
rf= lhs[,4]*(rf.max-rf.min)+rf.min,
nr= lhs[,5]*(nr.max-nr.min)+nr.min,
gf= lhs[,6]*(gf.max-gf.min)+gf.min,
cs= lhs[,7]*(cs.max-cs.min)+cs.min,
ca= lhs[,8]*(ca.max-ca.min)+ca.min,
p = lhs[,9]*(p.max-p.min)+p.min,
```

```
v = lhs[,10]*(v.max-v.min)+v.min,
k = lhs[,11]*(k.max-k.min)+k.min,
ep= lhs[,12]*(ep.max-ep.min)+ep.min,
e = lhs[,13]*(e.max-e.min)+e.min
)
###### treatment coverage n ####
l <- 40
########## simulating points ########
h2 <-500
### 13 varaibles + n + emergence = 15 ###
j <- 1
data <- data.frame(matrix(rep(NA,l*h2*15),nrow=l*h2))
for(i in 1:h2){
for (n in seq(0,1,length=l)){
xstart <- c(Is=1,P=0,Ir=0,Pr=0,Sh=623,Ims=0,Imr=0,Sm=2435)
params <- as.list(c(params.set[i,],n=n))
out <- as.data.frame(lsoda(xstart, times, falciP, params))
emergence<-(365*50-sum(as.numeric(out$Ir>out$Is)))/365
data[j,1:14] <- params
data[j,15] <- emergence
j <- j+1
}
}
names(data) <- c(names(params),'Emergence')
#### Sensitivity ####
require(sensitivity)
sad <- pcc(data[,1:14], data[,15], nboot = 100, rank=TRUE)
```

Figure A. Simulation of no-regimens

This figure illustrates the situation with no-regimen.

```
library(deSolve)
library(latex2exp)
time=seq(0,365*50)
  parf <- c(Nm=2435,Nh=624, a=0.21, b=0.5, sf=0.9, n=0, rf=1/287, nr=1, gf=1/2,cs=0.4,
            ca=0.12, mm=0.033,nm=0.033,p=0.95*0,k=30,ep=3,e=0.1,
            v=1/10e12)
  y=c(Is=1, P=0, Ir=0,Pr=0,Sh=623,Ims=0,Imr=0,Sm=2435) # initial condition
  OP <- ode(y,time,falciP,parf)
  years=OP[,1]/365
  par(mfrow=c(1,2))
  plot(years,OP[,6]/parf[2],xlim=c(min(years),max(years)),ylim=c(0,1),type="l",col="black"
       ,lwd=2,xlab="year",ylab="")
  lines(years,OP[,2]/parf[2],type="l",col="aquamarine4",lwd=2)#Is
  lines(years,OP[,3]/parf[2],type="l",col="aquamarine4", lty=4,lwd=2)#P
  lines(years,OP[,4]/parf[2],type="l",col="red",lwd=2)#Ir
  lines(years,OP[,5]/parf[2],type="l",col="red",lty=4,lwd=2)#Pr
  title("P. falciparum")
  legend("topright", legend=c(TeX('$S_h$'),TeX('$I_{fs}$'),
                              TeX('$P_{fs}$'),TeX('$I_{fr}$'),TeX('$P_{fr}$')),
         col=c("black","aquamarine4","aquamarine4","red","red"), lty=c(1,1,4,1,4), cex=1.1,
         lwd=2,ncol=1)
```

```
#### No-Regimen simulation function for P. vivax

  time=seq(0,365*50)
  parv <- c(Nm=2435,Nh=624,a=0.21,b=0.5,sv=0.33,n=0,rv=1/60,gv=1/9,cs=0.4,ca=0.12,
            mm=0.033,nm=0.033,p=0.95*0,v=1/10e12,k=3,ep=1.55,e=0.1,psi=1/60,
            psr=0.5,prs=0.5,phit=0.29,phiu=0.9,mvl=1/425,nr=1)
  y=c(Ivs=1, Lvs=0, Pv=0, Ivr=0, Lvr=0, Pvr=0, Sh=622, Imvs=0, Imvr=0, Sm=2435)
  OP <- ode(y,time,vivax,parv,method = "ode2")
  years=OP[,1]/365
  plot(years,OP[,8]/parv[2],xlim=c(min(years),max(years)),ylim=c(0,1),type="l",col="black"
       ,lwd=2,xlab="year",ylab="")
  lines(years,OP[,2]/parv[2],type="l",col="aquamarine4",lwd=2)#Ivs
  lines(years,OP[,3]/parv[2],type="l",col="aquamarine4",lwd=2,lty=2)#Lvs
  lines(years,OP[,4]/parv[2],type="l",col="aquamarine4",lwd=2,lty=4)#Pvs
  lines(years,OP[,5]/parv[2],type="l",col="red",lwd=2)#Ivr
  lines(years,OP[,6]/parv[2],type="l",col="red",lwd=2,lty=2)#Lvr
  lines(years,OP[,7]/parv[2],type="l",col="red",lwd=2,lty=4)#Pvr
  legend("topright", legend=c(TeX('$S_h$'),TeX('$I_{vs}$'),TeX('$L_{vs}$'),TeX('$P_{vs$}')
                             ,TeX('$I_{vr}$'),TeX('$L_{vr}$'),TeX('$P_{vr$}')),
         col=c("black","aquamarine4","aquamarine4","aquamarine4","red","red","red"),
         lty=c(1,1,2,4,1,2,4), cex=1.1,lwd=2,ncol=1)
  title("P. vivax")
```

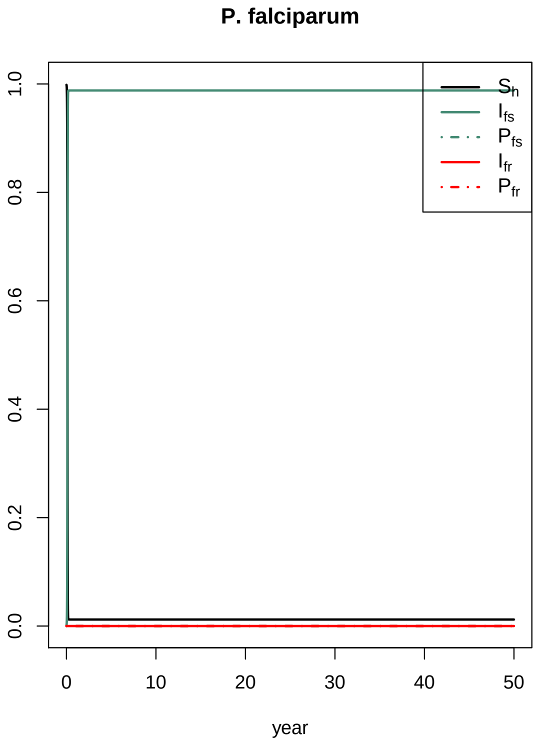

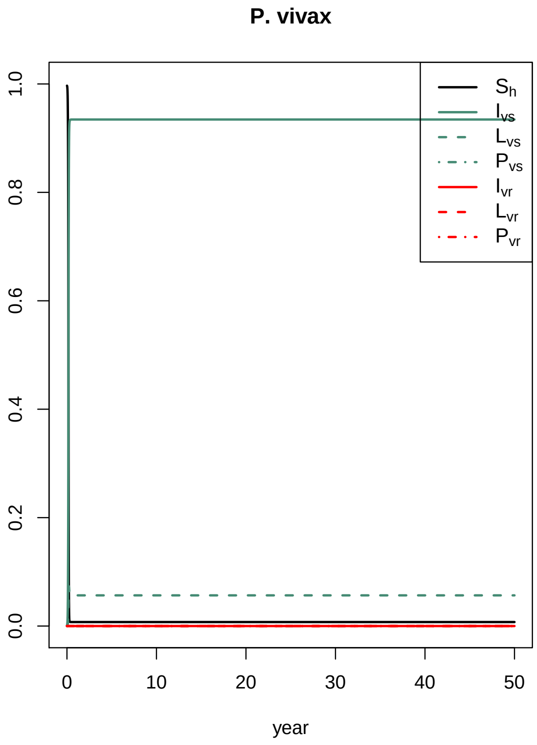

Supplement: S2 File — Document generated from code in S1—Code (in R language), parameters and data to reproduce simulation and figures. (PDF) [file pcbi.1007945.s002.pdf]
